# Unmet health care needs in immune thrombotic thrombocytopenic purpura survivors

**DOI:** 10.1016/j.rpth.2026.103461

**Published:** 2026-03-26

**Authors:** Charlotte M. Story, Jenna Brown, Clare Martin, Shruti Chaturvedi

**Affiliations:** 1 Division of Hematology, Department of Medicine, Johns Hopkins University School of Medicine, Baltimore, Maryland, USA; 2 United States Thrombotic Microangiopathy Alliance (USTMA), Groveport, Ohio, USA

**Keywords:** patient-reported outcome measures, purpura, surveys and questionnaires, thrombotic microangiopathy, thrombotic thrombocytopenic purpura

## Abstract

**Background:**

Immune thrombotic thrombocytopenic purpura (iTTP) is a rare thrombotic microangioathy episodic life-threatening thrombotic microangiopathy. Survivors of iTTP have high rates of chronic morbidities in remission such as depression, cognitive impairment, stroke, and increased cardiovascular mortality. Careful multidisciplinary management during clinical remission is needed to monitor for relapse and manage comorbidities, but this may not be universally available.

**Objectives:**

To identify gaps in the outpatient care of iTTP survivors.

**Methods:**

We conducted a cross-sectional survey-based study. The questionnaire included demographic information, disease-specific information, treatment-specific information, and the Healthcare Access and Utilization Survey developed by the National Institutes of Health All of Us Research Program.

**Results:**

A total of 281 participants responded to the survey. Median age was 47 years (IQR, 34–60 years). Most respondents were women (255/278, 91.7%), White (206/281, 73.7%), and highly educated with 215 of 263 (81.7%) reporting some college or further education. A quarter of respondents, 67 of 247 (27.1%), had not seen a hematologist in the past year, and 48 of 247 (19.4%) had not seen a primary care provider. Additionally, 45 of 279 (16.1%) reported not having an iTTP doctor. Laboratory monitoring was performed every 6 months or less frequently in 38% of respondents. Treatment for relapse prevention was not discussed with 54.8%. While 82.7% self-reported mental health disturbance after iTTP diagnosis, 30.4% had been referred to a mental health professional.

**Conclusion:**

In this United States-based cohort, despite high rates of medical and psychiatric comorbidities, only a minority reported high-quality relapse-prevention care. There were high rates of mental health disturbances after iTTP diagnosis but limited access to mental health care.

## Essentials

• Multimodal survivorship care is critical for thrombotic thrombocytopenic purpura survivors.
• This study surveyed patients regarding access to high-quality follow-up care and monitoring.
• More than 25% had not seen a hematologist >1 year; >35% reported delayed care for financial concerns.
• More than 80% reported new psychiatric concerns; there was limited access to mental health care.

## 1 Introduction

Thrombotic thrombocytopenic purpura (TTP) is a rare hematologic disorder caused by deficiency of the von Willebrand factor–cleaving protease ADAMTS-13, which leads to episodic thrombotic microangiopathy [1]. The more common form, immune thrombotic thrombocytopenic purpura (iTTP), is caused by acquired ADAMTS-13 antibodies. Without treatment, acute iTTP has a mortality of >90% [2]. Treatment with plasma exchange, immunosuppression, and caplacizumab has dramatically improved outcomes, and mortality is now <10%. Despite advances in therapy, the 5-year relapse risk of TTP has been reported to be ∼35% to 40% in recent studies and 30% to 50% or more in historical controls before rituximab therapy [[3], [4], [5], [6], [7]]. Recent research suggests that treatment of ADAMTS-13 relapse can reduce the risk of clinical relapse, underscoring the importance of ongoing laboratory monitoring and identification of partial ADAMTS-13 remission and ADAMTS-13 relapse [[8], [9], [10], [11]]. Expert recommendations now suggest clinical and laboratory monitoring every 3 months for ADAMTS-13 monitoring and considering pre-emptive immunosuppression for ADAMTS-13 relapse to prevent clinical relapse [12]. There is also an increasing recognition of the nonrelapse morbidity during iTTP remission, including neuropsychiatric sequalae, cognitive impairment, and cardiovascular disease [[13], [14], [15], [16], [17], [18], [19], [20], [21]]. We conducted this survey to evaluate the access to and quality of outpatient relapse—prevention and survivorship care in TTP survivors in the United States.

## 2 Methods

We performed a cross-sectional survey–based study. Survey tools were created in Research Electronic Data Capture (REDCap), a secure, web-based data collection and management platform hosted at Johns Hopkins Hospital. Eligible participants were adults aged ≥18 years, with a self-reported diagnosis of iTTP. The questionnaire included 4 sections as follows: (i) demographic information; (ii) TTP diagnosis and treatment with a focus on individuals’ experience with hematologic care, including relapse prevention, and appropriate referrals for mental health and other comorbidities; (iii) general health information including information on comorbidities such as hypertension, stroke, myocardial infarction, and depression, which are highly prevalent in TTP cohorts; and (iv) the Healthcare Access and Utilization Survey from the National Institutes of Health All of Us research program. The Healthcare Access and Utilization Survey instrument was chosen because it has been tested for readability and accessibility using cognitive interviews and quantitative testing in a testing process that included people from different educational backgrounds and geographic locations to capture a sample that reflects the US population. The survey link was distributed electronically via social media platforms (eg, Facebook and Twitter) and the United States Thrombotic Microangiopathy Alliance patient advocacy group email distribution lists from May 2022 until November 2022. Participation was voluntary, and responses were anonymous. Individuals provided electronic informed consent prior to initiating the survey.

Data were summarized using descriptive statistics. Differences between groups (between different self-reported race groups [Black, White, or other], insurance types [commercial, Medicare, Medicaid, or uninsured], and location [urban, suburban, or rural]) were compared using the chi-squared test or *t*-test for categorical and continuous variables, respectively. A *P* value of <.05 was considered significant. All analyses were performed using Stata software, version 18 (IBM Corp).

## 3 Results

### 3.1 Demographic information

There were 281 responses, of which 255 of 278 (91.7%) identified as from women. Median age was 47 years (IQR, 34–60 years). Most participants identified themselves as White (206/281, 73.3%) or Black (43/281, 15.3%). In terms of education attainment, 5 of 263 (1.9%) reported never attending the 12th grade, 41 of 263 (15.6%) completed high school or had a general educational development or equivalent, 35 of 263 (13.3%) had attended some college but not finished, 104 of 263 (39.5%) had an associate or bachelor’s degree, and 76 of 263 (28.9%) had a master’s degree or further education. Other self-reported sociodemographic information such as marital status, employment status, household composition, and geographic residence is summarized in Table 1. Unemployment due to TTP was reported in 15.8% of respondents.

**Table 1:** Respondent sociodemographic characteristics.

Variable	Value
Age (y; *n* = 277)	47 (34–60)
Sex: female (*n* = 278)	255 (91.7)
Race (*n* = 281)	
White	206 (73.3)
Black	43 (15.3)
Other	32 (11.4)
Highest educational attainment (*n* = 281)	
Never attended 12th grade	5 (1.9)
HS graduate	35 (13.3)
GED or equivalent	6 (2.3)
Some college	35 (13.3)
Associate/bachelor	104 (39.5)
Master’s degree or higher	76 (28.9)
Refused	0
Do not know	2 (0.8)
Marital status (*n* = 281)	
Married/couple	183 (65.8)
Divorced/separated/widow	34 (12.2)
Never married	56 (20.1)
Rather not say	5 (1.8)
Employment status (*n* = 240)	
Working full-time	133 (55.4)
Working part-time	32 (13.3)
Student	4 (1.7)
Homemaker	16 (6.7)
Unemployed	17 (7.1)
Unemployed due to TTP	38 (15.8)
What is your current income? (*n* = 242)	
$0–$50,000	108 (44.6)
$50–$100,000	56 (23.1)
$100,000–$150,000	19 (7.9)
>$150,000	11 (4.5)
Prefer not to answer	48 (19.8)
Adults in household (*n* = 281)	
Live alone	28 (9.9)
1 other adult	128 (45.6)
2 other adults	95 (33.8)
>3 other adults	30 (10.7)
Children in household (*n* = 268)	
No children	168 (62.7)
1 child	56 (20.9)
2 children	33 (12.3)
3 children	10 (3.7)
Urbanicity of residence (*n* = 279)	
Rural	66 (23.7)
Suburban	134 (48.0)
Urban	79 (28.3)

Values are *n* (%) or median (range).

GED, general educational development; HS, high school; TTP, thrombotic thrombocytopenic purpura.

### 3.2 Burden of post-TTP acquisition of new medical and psychiatric comorbidities

Participants reported comorbidities they may have experienced prior to or after iTTP. The responses are summarized in Table 2. Excluding patients with a pre-existing hypertension diagnosis, 40.4% of patients developed hypertension after TTP diagnosis. New stroke after TTP diagnosis occurred in 24.3% (54/222). Headache was common; of patients without prior headache diagnosis, 59.3% (80/135) developed new headache after TTP diagnosis. Of patients without pre-existing depression, 51.4% (95/185) received new depression diagnosis after their TTP diagnosis. Most participants (229/277, 82.7%), reported that they experienced depression or anxiety related to their iTTP. A minority (84/276, 30.4%), reported that they had been referred to a mental health professional. When asked whether their general health and well-being were better or worse after their iTTP diagnosis, most respondents reported their health was much worse (104/241, 43.2%) or slightly worse (93/241, 38.6%), and a minority reported their health was the same (32/241, 13.3%), slightly better (10/241, 4.1%) or much better (2/241, 0.8%).

**Table 2:** Participant-reported comorbidities and onset relative to TTP diagnosis.

Diagnosis	Total responses, *n*	No, never, *n* (%)	Yes, before TTP, *n* (%)	Yes, after TTP, *n* (%)
Hypertension	235	109 (46.4)	52 (22.1)	74 (31.5)
Myocardial infarction	213	199 (93.4)	5 (2.3)	9 (4.2)
Stroke	222	157 (70.7)	11 (5.0)	54 (24.3)
SLE	217	196 (90.3)	11 (5.1)	10 (4.6)
Headache	236	55 (23.3)	101 (42.8)	80 (33.9)
Depression	237	90 (38.0)	52 (21.9)	95 (40.1)

SLE, systemic lupus erythematosus; TTP, thrombotic thrombocytopenic purpura.

### 3.3 Experiences with TTP care

**Figure 1:**
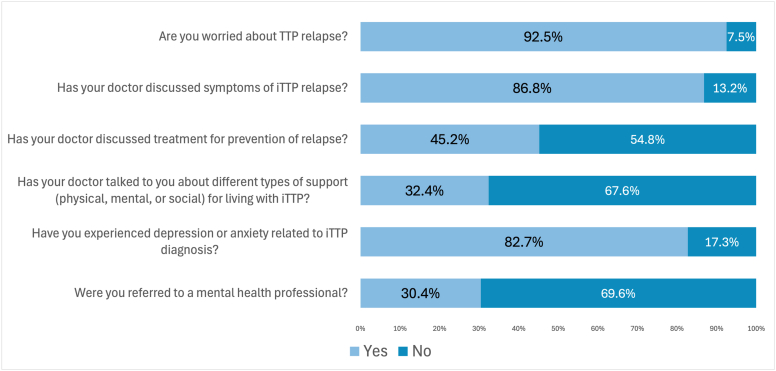
Unmet needs in preventive care for patients with iTTP. iTTP, immune thrombotic thrombocytopenic purpura; TTP, thrombotic thrombocytopenic purpura.

Most participants (234/279, 83.9%) indicated being under the care of a TTP specialist, and 229 of 279 (82.9%), reporting feeling that their doctor is knowledgeable about iTTP. However, 16.1% (45/279) of patients reported not having a TTP doctor. A majority reported that their doctor had discussed risk of relapse (223/279, 79.9%), symptoms of TTP relapse (244/281, 86.8%), and next steps in the event of relapse (231/280, 82.5%). Moreover, 45.2% (126/279) of patients reported that their doctor discussed treatment to prevent relapses. Most participants (260/281, 92.5%), reported that they were worried about an iTTP relapse (Figure 1). While most participants felt that their doctor listened to their concerns always or often (167/276, 60.5%), a minority (90/278, 32.4%) reported that their doctor talked to them about types of support (mental, physical, or social) for living with iTTP (Figure 1). In terms of distance to care, 36 of 275 participants (13.1%) reported a 0- to 10-minute commute to their hematologists’ office, while 98 of 275 (35.6%) reported 10 to 30 minutes, 65 of 275 (23.6%) reported 30 minutes to an hour, and 76 of 275 (27.6%) reported >1 hour distance to hematologic care.

Most participants (249/278, 89.6%) indicated that their TTP care included undergoing routine blood work. Frequency of blood work varied, with 86 of 276 (31.2%) reporting more than once every 3 months, 85 of 276 (30.8%) going once every 3 months, 48 of 276 (17.4%) going every 6 months, and 57 of 276 (20.7%) having blood workup done ≤1 time a year.

### 3.4 Healthcare Access and Utilization Survey

Regarding the health care access and utilization section of the survey, 54.3% (133/245) of participants had private insurance, 20.4% (50/245) had Medicaid or Medicare, (18/245) 7.3% did not have health insurance in the past year, and 17.9% (44/245) reported other or prefer not to say (Table 3). In the past year, 13.2% (32/242) of participants were told that their insurance would not cover a health care service. Compared with a year earlier, 14.6% (35/239) of participants reported worse health insurance coverage, 77.4.% (185/239) reported similar coverage, and 7.9% (25/239) reported that their coverage was better.

**Table 3:** Healthcare Access and Utilization Survey: insurance status, financial toxicity, and experiences with medical care.

Variable	Value, *n* (%)
Insurance (*n* = 245)	
None	18 (7.3)
Private insurance	133 (54.3)
Medicare	34 (13.9)
Medicaid	16 (6.5)
Other	39 (15.9)
Prefer not to say	5 (2.0)
In the past year, were you told your insurance was not covered? (*n* = 242)	
Yes	32 (13.2)
How does coverage compare to a year ago? (*n* = 239)	
Better	19 (7.9)
Worse	35 (14.6)
About the same	185 (77.4)
Has there been a time when you needed something and could not get it? (*n* = 242)	
Prescription medicine	41 (16.9)
Mental health care	37 (15.3)
Emergency room care	15 (6.2)
Dental care	49 (20.2)
Eyeglasses	39 (16.1)
To see a regular doctor	19 (7.9)
To see specialist	32 (13.2)
Follow-up	24 (9.9)
How worried about you about paying your medical bills? (*n* = 244)	
Very worried	63 (25.8)
Moderately worried	56 (23.0)
Slightly worried	54 (22.1)
Not worried at all	71 (29.1)
Have you skipped medication to save money? (*n* = 243)	
Yes	34 (14.0)
Have you delayed a prescription to save money? (*n* = 243)	
Yes	38 (15.6)
Have you gotten medicine from another country? (*n* = 243)	
Yes	4 (1.6)
Have you used alternative therapies to save money? (*n* = 242)	
Yes	35 (14.5)
Is there a place you go when you need medical care? (*n* = 245)	
Yes	202 (82.4)

**Figure 2:**
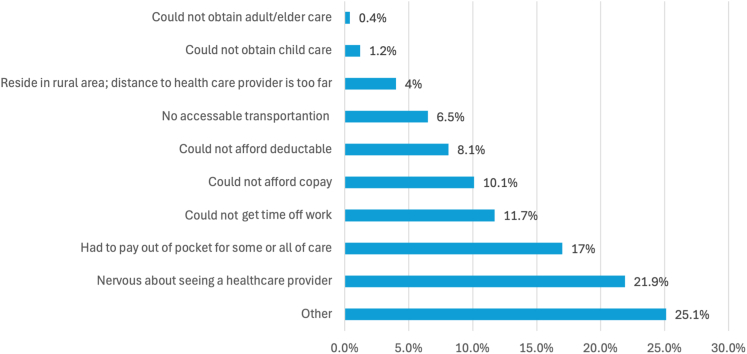
Patient-reported factors contributing to delays in medical care.

In terms of financial barriers to care (Table 3), 48.8% (119/244) of respondents reported being very or moderately worried about paying their medical bills, compared with 22.1% (54/244) who were slightly worried and 29.1% (71/244) who were not worried at all. Skipped doses or delayed doses of medication to save money were reported by 14% (34/243) and 15.6% (38/243) of respondents, respectively. Turning to alternative therapies to save money was reported by 14.5% (35/242) of respondents. A summary of reasons for delay in medical care is provided in Figure 2; 35.2% (87/247) report delaying care due to issues with payment, including high deductibles (8.1%), unaffordable copay (10.1%), and paying out-of-pocket (17.0%).

Geographic differences and transportation responses are summarized in Table 4. Most participants (189/232, 81.4%) reported last contact with a health care provider within the past 0 to 3 months of taking the survey, followed by 9.5% (22/232) within 4 to 6 months, and 9.1% (21/232) reported last contact more than 6 months prior. Furthermore, 80.6% (199/247) reported seeing a primary care or general doctor and 72.9% (180/247) reported seeing a hematologist within the last year. Within the last year, 26.7% (66/247) reported seeing a mental health professional.

**Table 4:** Healthcare Access and Utilization Survey: geography and transportation.

Variable	Value, *n* (%)
What type of area is your health care provider? (*n* = 244)	
Urban	115 (47.1)
Suburban	103 (42.2)
Rural	26 (10.7)
How long does it take for you to get to your health care provider? (*n* = 246)	
0–10 min	54 (22.0)
10–30 min	131 (53.3)
30 min to 1 h	37 (15.0)
>1 h	24 (9.8)
How long since you talked to a provider about your health? (*n* = 232)	
0–3 mo	189 (81.4)
4–6 mo	22 (9.5)
7–9 mo	1 (0.4)
10 mo to 1 y	13 (5.6)
>1 y	7 (3.0)
Which professionals have you talked to in the last year? (*n* = 247)	
Hematologist	180 (72.9)
General care doctor	199 (80.6)
NP, PA, midwife	109 (44.1)
ObGYN	62 (25.1)
Mental health professor	66 (26.7)
Eye doctor	114 (46.2)
Podiatrist	17 (6.9)
Chiropractor	29 (11.7)
PT, ST, OT	32 (13.0)
Dentist	115 (46.6)
Healer	13 (5.3)
Has there been a time when you needed something and could not get it? (*n* = 242)	
Prescription medicine	41 (16.9)
Mental health care	37 (15.3)
Emergency room care	15 (6.2)
Dental care	49 (20.2)
Eyeglasses	39 (16.1)
To see a regular doctor	19 (7.9)
To see specialist	32 (13.2)
Follow-up	24 (9.9)

Responses regarding quality of care, communication, and similarity between patient and provider are summarized in Table 5. Most participants (226/247, 91.5%) reported that they were treated with respect by their doctor most of or all of the time. Approximately half (123/243, 50.6%) also reported that they were asked their opinions or beliefs about their health care all or most of the time, but 19.8% (48/243) reported this never happened.

**Table 5:** Healthcare Access and Utilization Survey: quality of care, communication, and similarity with health care provider.

Survey question	All of the time (%)	Most of the time (%)	Some of the time (%)	Never (%)
How often were you treated with respect by your provider? (*n* = 247)	61.5	30.0	7.7	0.8
How often did your doctors or health care providers ask your opinions or beliefs about your medical care or treatment? (*n* = 243)	21.4	29.2	29.6	19.8
How often did your doctors or health care providers tell or give you information about your health and health that was easy to understand? (*n* = 247)	36.4	45.3	16.6	1.6
	Very important	Moderately important	Slightly important	Not important at all
How important is it to you that your doctors or health care providers understand or are similar to you in any of these ways (racially, ethnically, financially, spiritually, interests)?	21.9	18.2	21.1	38.8
	Very often	Sometimes	Not often	Never
How often were you able to see doctors or health care providers who were similar to you in any of these ways?	32.9	36.8	18.6	11.7
How often have you either delayed or not gone to see doctors or health care providers because they were different from you in any of these ways?	1.6	9.0	15.2	74.2

Participant perspective varied regarding the importance of provider similarity (racially, ethnically, financially, spiritually, or interests), with 21.9% (53/242) finding it very important, 18.2% (44/242) finding it moderately important, 21.1% (51/242) finding it slightly important, and 38.8% (94/242) finding it not important at all (Table 5). Regarding frequency that participants saw providers who were similar to them, 32.9% (76/231) reported very often, 36.8% (85/231) reported sometimes, 18.6% (43/231) reported not often, and 11.7% (27/231) reported never.

A comparison of health care experiences and access to care across geographic location, insurance status, and race was performed (Supplementary Tables 1–16). These analyses were limited by the small number of respondents identifying as non-White and by limited representation across insurance types and residential settings. Although some statistically significant differences were observed, the potential for type I error is high given the number of comparisons performed and the small sample size. The supplementary tables also show for all responses from the health care access and utilization survey, including data not presented.

## 4 Discussion

With improved survival of acute iTTP episodes, there is an increased need for survivorship care focused both on relapse prevention and managing risk of stroke and cardiovascular disease. There is also a need to address the mental health and cognitive challenges that are a major concern for patients. Despite the fact that nearly all respondents report concern about relapse risk and recent expert recommendations on the management of TTP in remission, this survey of patient experiences and access to relapse-prevention care found that less than half of patients are provided guidance regarding relapse prevention (including preemptive immunosuppression for ADAMTS-13 relapse), and >20% receive clinical and laboratory monitoring once a year or less frequently.

This survey confirms previous findings that relapses are a leading concern for iTTP survivors. It also confirms the high burden of new comorbidity acquisition, including hypertension, stroke, myocardial infarction, lupus, and psychiatric disorders such as depression diagnosed following the diagnosis of TTP. In total, 82.7% of respondents self-reported new concerns with anxiety or depression related to their TTP diagnosis. Of those without pre-existing depression, 40% reported a new depression diagnosis after iTTP diagnosis, which is in contrast to the less than one-third who were offered referral for mental health or other support or resources.

These findings are particularly concerning since respondents in our study were predominantly highly educated White women with college degrees. There is consistent evidence that male sex, younger age, disadvantaged socioeconomic status, and lower educational attainment are associated with lower survey response rate [[22], [23], [24]]. Importantly, several studies note lower response rates in non-White individuals in the United States [24,25]. The predominance of female respondents is expected, as the incident rate ratio for iTTP in women compared with that of men is 3.19 (95% CI, 2.65–3.85) [1]. However, Black individuals are disproportionately impacted by both higher incidence of iTTP (incident rate ratio, 7.09; 95% CI, 6.05–8.31) and adverse health outcomes of iTTP [1,4]. Moreover, individuals who are subscribed to email lists or social media pages of a TTP patient support organization are more likely to be highly engaged in their health care and self-advocate. Thus, due to sampling error and responder bias, our study likely grossly underestimates gaps in preventive care for all individuals living with iTTP.

Our findings highlight pervasive challenges that impact access to high-quality care for rare diseases spanning access to care, care coordination, and providers’ knowledge gaps. Barriers to care identified by survey respondents included major financial and insurance barriers, with 35.2% reporting delaying care due to issues with payment, including high deductibles, unaffordable copays, and necessity of paying out-of-pocket. Survivorship care in TTP requires specialized diagnostics, ongoing monitoring, and additional treatment for relapses, which can expose patients to financial toxicity in the current American Health Insurance paradigm where coverage does not guarantee financial protection. It is striking that 48.8% of respondents expressed being moderately or very worried about paying their medical bills, underscoring the immense financial toxicity of this disease. Other barriers, including insufficient transportation, need for time off work, and lack of child, adult, and elder care were also reported. Considering >80% of patients reported anxiety and depression related to iTTP diagnosis and 92.5% reported worry of relapse, it is not surprising that 21.9% reported health care anxiety as a barrier to care, highlighting the need for trauma-informed care among clinicians who care for this patient population.

While not fully elicited in this survey, the discrepancy between patients with access to hematologic care (72.9% reported seeing a hematologist in the last year) and those receiving comprehensive relapse-prevention counseling or appropriate referrals to supportive services is likely at least partially attributable to knowledge and awareness gaps among both patients and health care providers. Insufficient rare disease education. Among the current health care workforce limits our ability to efficiently tackle disease-specific challenges, and there is a scarcity of clinical expertise as rare diseases affect small populations with geographically scattered patients [26]. A lack of multidisciplinary care frameworks, which are essential for the comprehensive care of a disorder with wide ranging multisystem sequelae, further impedes optimal outcomes. Patient advocacy organizations can play an important role in providing patient-centered education that helps patients navigate treatment decisions and challenges with access to care, as well as by promoting awareness among health care providers. Advocacy groups can be critical for breaking down barriers to care. One example is the free ADAMTS-13 testing program offered by the United States Thrombotic Microangiopathy Alliance. In the rare disease landscape, reference or expert centers can also improve outcomes by providing access to specialized care through virtual case conferences, disease registries, and access novel therapies through clinical trials.

To conclude, in this United States–based survey of iTTP survivors, there was a high rate of new medical and psychiatric comorbidities after diagnosis of iTTP. Financial barriers to care were common, and approximately a third of respondents reported delaying care due to financial constraints. There were low reported rates of high-quality relapse prevention care, and 27.1% had not seen a hematologist in the past year. Given that were predominantly White women with high degree of educational attainment, these findings likely grossly underestimate the degree of barrier to care in populations with more socioeconomic disadvantage. Future studies are needed to evaluate health care access and experiences in more socioeconomically, educationally, and racially diverse populations to adequately capture the full scope of barriers to care among iTTP survivors. As this is a United States–based study, studies in international populations are needed to assess generalizability in populations with substantially different health care and insurance structures.
