# Supplementary material for: Unmet health care needs in immune thrombotic thrombocytopenic purpura survivors
Source: Res Pract Thromb Haemost. 2026 Mar 26;10(3):103461. doi: 10.1016/j.rpth.2026.103461 (PMC13156633; doi:10.1016/j.rpth.2026.103461)
Supplement: Supplementary Material [file mmc1.docx]

**Supplemental Data**

**Aggregate responses to all survey questions.**

Table 1: Aggregate responses to survey: Sociodemographic information

| Category | Total Cohort |
| --- | --- |
| Age Median (IQR) | 47 (34-60)  Total: 277 |
| Sex (female, %) | 255/278 (91.7%) |
| Race (%)  White  Black  Other | Total: 281  206 (73.3%)  43 (15.3%)  32 (11.4%) |
| Highest degree of school  Never attended 12th  HS Graduate  GED or Equivalent  Some college  Associate/ Bachelor  Master's and higher  Refused  Don’t know | Total: 263  5(1.9%)  35 (13.3%)  6 (2.3%)  35 (13.3%)  104 (39.5%)  76 (28.9%)  0  2 (0.8%) |
| Marital Status (%)  Married/ Couple  Divorced/Sep/widow  Never married  Rather not say | Total: 278  183 (65.8%)  34 (12.2%)  56 (20.1%)  5 (1.8%) |
| Adults in household  Live alone  One other adult  Two other adults  Three or more other adults | Total: 281  28 (9.9%)  128 (45.6%)  95 (33.8%)  30 (10.7%) |
| Children in household  No children  One child  Two children  Three children | Total: 268  168 (62.7%)  56 (20.9%)  33 (12.3%)  10 (3.7%) |
| Type of living (%)  Rural  Sub-urban  Urban | Total: 279  66 (23.7%)  134 (48.0%)  79 (28.3%) |

Table 2: Aggregate responses to questions regarding TTP diagnosis, therapy

| Category | Total |
| --- | --- |
| Year of TTP dx Median (IQR) | 2013 (8 yrs)  Total: 280 |
| TTP Episodes Median (IQR) | 1(3)  Total: 275 |
| Are you worried about relapse? (%) | 260/281  (92.5%) |
| Has your doctor discussed symptoms of TTP relapse? (%) | 244/281  (86.8 %) |
| Has your doctor discussed what to do in a relapse? (%) | 231/280 (82.5%) |
| Treatments received  Steroids  Prednisone  Plasma Exchange  Rituximab  Caplacizumab  Other  I don’t know | Total: 281  179 (63.7%)  216 (76.9%)  275 (97.9%)  209 (74.4%)  49 (17.4%)  61 (21.7%)  2 (0.7%) |
| Has your doctor discussed the risk of TTP relapse? (%) | 223/279 (79.9%) |
| Has your doctor discussed treatment for relapse prevention? (%) | 126/279 (45.2%) |
| Do you have a TTP doctor? (%) | 234/279  (83.9%) |
| Do you feel your doctor is knowledgeable about TTP? (%) | 229/277 (82.6%) |
| How long does it take to get to your doctor? (%)  0-10 minutes  10-30 minutes  30 minutes- hour  Over 1 hour | Total: 275    36 (13.1%)  98 (35.6%)  65 (23.6%)  76 (27.6%) |
| Type of TTP DR (%)  Hematologist  Heme/Onc  Other/Don’t know | Total: 276  90 (32.6%)  166 (60.1%)  20 (7.2%) |
| Blood Work (%) | 249/278 (89.6%) |
| Freq of labs (%)  More than 3 mo  3 mo  6 mo  1 yr  Less than 1 year | Total: 276  86 (31.2%)  85 (30.8%)  48 (17.4%)  33 (12.0%)  24 (8.7%) |
| Type of Labs (%)  CBC/Platelets  LDH  ADAMTS13  Unsure | Total: 281  264 (93.9%)  193 (68.7%)  181 (64.4%)  17 (6.0%) |
| Do you feel your doctor listens to your concerns (%)  Always  Often  Sometimes  Never | Total: 276    167 (60.5%)  55 (19.9%)  46 (16.7%)  8 (2.9%) |
| Did your doctor discuss types of support for living with TTp?  Yes | 90/278 (32.4%) |
| Concerns for Depression or Anxiety  Yes | 229/277 (82.7%) |
| Referred to Mental health professional (%) | 84/276 (30.4%) |

Table 3: Aggregate Responses to Healthcare access and Utilization Survey

| Category | Total |
| --- | --- |
| Insurance (%)  None  Private insurance  Medicare  Medicaid  Other  Prefer not to say | Total: 245  18 (7.3%)  133 (54.3%)  34 (13.9%)  16 (6.5%)  39 (15.9%)  5 (2.0%) |
| In the past year, were you told your insurance was not covered? | 32/242 (13.2%) |
| How does coverage compare to a year ago?  Better  Worse  About the same | Total: 239  19 (7.9%)  35 (14.6%)  185 (77.4%) |
| Is there a place you go when you need medical care? | 202/245 (82.4%) |
| What type of area is your health care provider?  Urban  Suburban  Rural | Total: 244  115 (47.1%)  103 (42.2%)  26 (10.7%) |
| How long does it take for you to get to your healthcare provider?  0-10 mins  10-30 mins  30 – 1 hour  Over 1 hour | Total: 246  54 (22.0%)  131 (53.3%)  37 (15.0%)  24 (9.8%) |
| How long since you talked to a provider about your health?  0-3 months  4-6 months  7-9 months  10 months – 1 year  >1 year | Total: 232  189 (81.4%)  22 (9.5%)  1 (0.4%)  13 (5.6%)  7 (3.0%) |
| Which professionals have you talked to in the last year?  Hematologist  General care doctor  NP,PA, midwife  ObGYN  Mental Health Prof  Eye doctor  Podiatrist  Chiropractor  PT, ST, OT  Dentist  Healer | Total: 247  180 (72.9%)  199 (80.6%)  109 (44.1%)  62 (25.1%)  66 (26.7%)  114 (46.2%)  17 (6.9%)  29 (11.7%)  32 (13.0%)  115 (46.6%)  13 (5.3%) |
| How often were you treated with respect by your provider?  All of the time  Most of the time  Some of the time  Never | Total: 247  152 (61.5%)  74 (30.0%)  19 (7.7%)  2 (0.8%) |
| How often were you asked about your beliefs on healthcare?  All of the time  Most of the time  Some of the time  Never | Total: 243  52 (21.4%)  71 (29.2%)  72 (29.6%)  48 (19.8%) |
| How often was information from your doctor easy to understand?  All of the time  Most of the time  Some of the time  Never | Total: 247  90 (36.4%)  112 (45.3%)  41 (16.6%)  4 (1.6%) |
| Have you delayed medical care for any of the following reasons?  No transportation  Rural  Nervous  No time off work  No childcare  No adult care  Couldn’t afford copay  Deductible too high  Pay out of pocket  Other | Total: 247  16 (6.5%)  10 (4.0%)  54 (21.9%)  29 (11.7%)  3 (1.2%)  1 (0.4%)  25 (10.1%)  20 (8.1%)  42 (17.0%)  62 (25.1%) |
| What is your employment status?  Working full time  Working part time  Student  Homemaker  Unemployed  Unemployed bc of TTP | Total: 240  133 (55.4%)  32 (13.3%)  4 (1.7%)  16 (6.7%)  17 (7.1%)  38 (15.8%) |
| What is your current income?  0-50k  50-100k  100-150k  More than 150k  Prefer not to answer | Total: 242  108 (44.6%)  56 (23.1%)  19 (7.9%)  11 (4.5%)  48 (19.8%) |
| Has there been a time when you needed something and could not get it?  Prescription medicine  Mental Health care  ER care  Dental care  Eyeglasses  To see a reg doctor  To see specialist  Follow up | Total: 242  41 (16.9%)  37 (15.3%)  15 (6.2%)  49 (20.2%)  39 (16.1%)  19 (7.9%)  32 (13.2%)  24 (9.9%) |
| How worried about you about paying your medical bills?  Very worried  Moderately worried  Slightly worried  Not worried at all | Total: 244  63 (25.8%)  56 (23.0%)  54 (22.1%)  71 (29.1%) |
| Have you skipped medication to save money? | 34/243 (14.0%) |
| Have you delayed a prescription to save money? | 38/243 (15.6%) |
| Have you gotten medicine from another country? | 4/243 (1.6%) |
| Have you used alternative therapies to save money? | 35/242 (14.5%) |
| Importance of provider similarity /understanding  Very important  Moderately important  Slightly important  Not at all | Total: 242  53 (21.9%)  44 (18.2%)  51 (21.1%)  94 (38.8%) |
| How often were you able to see providers who were similar to you?  Very often  Sometimes  Not often  Never | Total: 231  76 (32.9%)  85 (36.8%)  43 (18.6%)  27 (11.7%) |
| How often have you delayed care due to differences?  Very often  Sometimes  Not often  Never | Total: 244  4 (1.6%)  22 (9.0%)  37 (15.2%)  181 (74.2%) |

Table 4: Aggregate Responses to Comorbidities Questions

| Category | Total |
| --- | --- |
| Hypertension  No, never  Yes, before TTP  Yes, after TTP | Total: 235  109 (46.4%)  52 (22.1%)  74 (31.5%) |
| Lupus  No, never  Yes, before TTP  Yes, after TTP | Total: 217  196 (90.3%)  11 (5.1%)  10 (4.6%) |
| Heart Attack  No, never  Yes, before TTP  Yes, after TTP | Total: 213  199 (93.4%)  5 (2.3%)  9 (4.2%) |
| Stroke  No, never  Yes, before TTP  Yes, after TTP | Total: 222  157 (70.7%)  11 (5.0%)  54 (24.3%) |
| Headaches  No, never  Yes, before TTP  Yes, after TTP | Total: 236  55 (23.3%)  101 (42.8%)  80 (33.9%) |
| Depression  No, never  Yes, before TTP  Yes, after TTP | Total: 237  90 (38.0%)  52 (21.9%)  95 (40.1%) |
| Do you believe your life is better?  Much worse  Slightly worse  The same  Slightly better  Much better | Total: 241  104 (43.2%)  93 (38.6%)  32 (13.3%)  10 (4.1%)  2 (0.8%) |

**Analysis for differences by self-reported race**

Table 5: Differences in sociodemographic information by race

| Category | Total Cohort | White | Black | Other | P value |
| --- | --- | --- | --- | --- | --- |
| Age Median (IQR) | 47 (28-66)  Total: 281 | 48 (28-68)  Total: 208 | 46.5(29.5-63.5)  Total: 43 | 46 (34.5-57.5)  Total: 32 | Black: 0.476  White: 0.311 |
| Sex (female, %) | 255/278 (91.4%) | 187/204 (91.7%) | 42/43 (97.7%) | 26/31 (83.9%) | 0.104 |
| Highest degree of school  Never attended-12th  HS Graduate  GED or Equivalent  Some college  Associate/ Bachelor  Master's and higher  Refused  Don’t know | Total: 263    5(1.9%)  35 (13.3%)  6 (2.3%)  35 (13.3%)  104 (39.5%)  76 (28.9%)  0  2 (0.8%) | Total: 194    5 (2.6%)  29 (14.9%)  5 (2.6%)  20 (10.3%)  82 (42.3%)  51 (26.3%)  0  2 (1.0%) | Total: 40    0  1 (2.5%)  1 (2.5%)  8 (20.0%)  13 (32.5%)  17 (42.5%)  0  0 | Total: 29    0  5 (17.2%)  0  7 (24.1%)  9 (31.0%)  8 (27.6%)  0  0 | 0.553 |
| Marital Status (%)  Married/ Couple  Divorced/Sep/widow  Never married  Rather not say | Total: 278  183 (65.8%)  34 (12.2%)  56 (20.1%)  5 (1.8%) | Total: 205  154 (75.1%)  21 (10.2%)  28 (13.7%)  2 (0.9%) | Total: 42  11 (26.2%)  10 (23.8%)  20 (47.6%)  1 (2.6%) | Total: 31  18 (58.6%)  3 (9.7%)  8 (25.8%)  2 (6.5%) | <0.001 |
| Adults in home Median (IQR) | 1 (0)  Total: 253 | 1 (0-2)  Total: 208 | 1(0-2)  Total: 43 | 2 (0-4)  Total: 32 | White: 0.321  Black: 0.476 |
| Children in home median (IQR) | 1(0)  Total: 268 | 0(0-1)  Total:208 | 0(0-1)  Total: 43 | 0.5 (0-1.25)  Total: | White: 0.124  Black: 0.895 |
| Type of living (%)  Rural  Sub-urban  Urban | Total: 279  66 (23.7%)  134 (48.0%)  79 (28.3%) | Total: 205  58 (28.3%)  93 (45.4%)  54 (26.3%) | Total: 43  3 (7.0%)  31 (72.1%)  9 (20.9%) | Total: 31  5 (16.1%)  10 (32.3%)  16 (51.6%) | <0.001 |

Table 6: Differences in TTP diagnosis, therapy history by race

| Category | Total | White | Black | Other | P value |
| --- | --- | --- | --- | --- | --- |
| Year of TTP dx Median (IQR) | 2015 (10yrs)  Total: 280 | 2015 (10yrs)  Total: 208 | 2017(7yrs)  Total: 43 | 2017(11yrs)  Total: 32 | White: 0.240  Black: 0.236 |
| TTP Episodes Median (IQR) | 1(3)  Total: 275 | 1(3)  Total:208 | 2(2.75)  Total:43 | 1 (2.25)  Total: 32 | White: 0.263  Black: 0.316 |
| Are you worried about relapse? (%) | 260/281  (92.5%) | 190/206 (92.2%) | 38/43 (88.4%) | 32/32  (100.0%) | 0.159 |
| Has your doctor discussed symptoms of TTP relapse? (%) | 244/281  (86.8 %) | 177/206 (85.9%) | 37/43 (86.0%) | 30/32 (93.8%) | 0.470 |
| Has your doctor discussed what to do in a relapse? (%) | 231/280 (82.5%) | 173/205 (84.4%) | 31/43 (72.1%) | 27/32 (84.4%) | 0.149 |
| Treatments  Steroids  Prednisone  Plasma Exchange  Rituximab  Caplacizumab  Other  I don’t know | Total: 281  179 (63.7%)  216 (76.9%)  275 (97.9%)  209 (74.4%)  49 (17.4%)  61 (21.7%)  2 (0.7%) | Total: 206  127 (61.7%)  162 (78.6%)  201 (97.6%)  149(72.3%)  34(16.5%)  49(23.8%)  1(0.5%) | Total: 43  29 (67.4%)  30 (69.8%)  42 (97.7%)  35 (81.4%)  5 (11.6%)  8 (18.6%)  0 (0.0%) | Total: 32  23 (71.9%)  24 (75.0%)  32 (100.0%)  25 (78.1%)  10 (31.3%)  4 (12.5%)  1 (3.1%) | 0.459  0.439  0.674  0.407  0.068  0.307  0.213 |
| Has your doctor discussed the risk of TTP relapse? (%) | 223/279 (79.9%) | 168/204 (82.4%) | 31/43 (72.1%) | 24/32 (75.0%) | 0.237 |
| Has your doctor discussed tx for relapse prevention?  (%) | 126/279 (45.2%) | 85/204 (41.7%) | 25/43 (58.1%) | 16/32 (50.0%) | 0.120 |
| Do you have a TTP doctor? (%) | 234/279  (83.9%) | 166/204 (81.4%) | 39/43 (90.1%) | 29/32 (90.6%) | 0.174 |
| Do you feel your doctor is knowledgeable about TTP? (%) | 229/277 (82.6%) | 167/203  (82.0%) | 34/42 (81.0%) | 28/32  (87.5%) | 0.730 |
| How long does it take to get to your doctor? (%)  0-10 minutes  10-30 minutes  30 minutes- hour  Over 1 hour | Total: 275      36 (13.1%)  98 (35.6%)  65 (23.6%)  76 (27.6%) | Total: 200      27 (13.5%)  66 (33.0%)  49 (24.5%)  58 (29.0%) | Total: 43      7 (31.2%)  23 (53.5%)  7 (31.2%)  6 (14.0%) | Total: 32      2 (6.3%)  9 (28.1%)  9 (28.1%)  12 (37.5%) | 0.068 |
| Type of TTP DR (%)  Hematologist  Heme/Onc  Other/Don’t know | Total: 276  90 (32.6%)  166 (60.1%)  20 (7.2%) | Total: 202  70 (34.7%)  118 (58.4%)  14 (6.9%) | Total: 42  11 (26.2%)  29 (69.0%)  2 (4.8%) | Total: 32  9 (9.4%)  19 (59.4%)  4 (12.5%) | 0.517 |
| Blood Work (%) | 249/278 (89.6%) | 177/203 (87.2%) | 41/43 (95.3%) | 31/32 (96.9%) | 0.101 |
| Freq of labs (%)  More than 3 mo  3 mo  6 mo  1 yr  Less than 1 year | Total: 276  86 (31.2%)  85 (30.8%)  48 (17.4%)  33 (12.0%)  24 (8.7%) | Total: 202  63 (31.2%)  56 (27.7%)  37 (18.3%)  26 (12.9%)  20 (9.9%) | Total: 42  11 (26.2%)  17 (40.4%)  7 (16.7%)  4 (9.5%)  3 (7.1%) | Total: 32  12 (37.5%)  12 (37.5%)  4 (12.5%)  3 (9.4%)  1 (3.1%) | 0.671 |
| Type of Labs (%)  CBC/Platelets  LDH  ADAMTS13  IDK | Total: 281  264 (93.9%)  193 (68.7%)  181 (64.4%)  17 (6.0%) | Total: 206  192 (93.2%)  136 (66.0%)  128 (62.1%)  14 (6.7%) | Total:43  42 (97.7%)  32 (74.4%)  29 (67.4%)  0 (0.0%) | Total: 32  30 (93.8%)  25 (78.1%)  24 (75.0%)  3 (9.4%) | 0.534  0.264  0.332  0.166 |
| Do you feel your doctor listens to your concerns (%)  Always  Sometimes  Often  Never | Total: 276      167 (60.5%)  55 (19.9%)  46 (16.7%)  8 (2.9%) | Total: 201      123 (61.2%)  40 (19.9%)  33 (16.4%)  5 (2.5%) | Total: 43      23 (41.5%)  10 (23.2%)  8 (18.6%)  2 (4.7%) | Total: 32      21 (65.6%)  5 (15.6%)  5 (15.6%)  1 (3.1%) | 0.942 |
| Types of support (%) | 90/278 (32.4%) | 65/203 (32.0%) | 14/43 (32.6%) | 11/32 (34.4%) | 0.965 |
| Depression or Anxiety (%) | 229/277 (82.7%) | 164/203 (80.8%) | 35/42 (83.3%) | 30/32 (93.8%) | 0.196 |
| Referred to Mental health professional (%) | 84/276 (30.4%) | 63/202 (31.2%) | 13/43 (30.2%) | 8/31 (25.8%) | 0.832 |

Table 7: Differences in Healthcare Access and Utilization Survey by Race

| Category | Total | White | Black | Other | P value |
| --- | --- | --- | --- | --- | --- |
| Insurance (%)  None  Private insurance  Medicare  Medicaid  Other  Prefer not to say | Total: 245  18 (7.3%)  133 (54.3%)  34 (13.9%)  16 (6.5%)  39 (15.9%)  5 (2.0%) | Total: 178  9 (3.2%)  100 (56.2%)  30 (16.9%)  10 (5.6%)  26 (14.6%)  3 (1.7%) | Total: 39  5 (12.8%)  19 (48.7%)  4 (10.2%)  3 (7.7%)  7 (17.9%)  1 (2.6%) | Total: 28  4 (14.3%)  14 (50.0%)  0 (0.0%)  3 (10.7%)  6 (21.4%)  1 (3.8%) | 0.230 |
| In the past year, were you told your insurance was not covered? | 32/242 (13.2%) | 19/175 (10.9%) | 7/39 (17.9%) | 6/28 (21.4%) | 0.196 |
| How does coverage compare to a year ago?  Better  Worse  About the same | Total: 239      19 (7.9%)  35 (14.6%)  185 (77.4%) | Total: 172      11 (6.4%)  22 (12.8%)  139 (80.8%) | Total: 39      5 (12.8%)  7 (17.9%)  27 (69.2%) | Total: 28      3 (10.7%)  6 (21.4%)  19 (67.9%) | 0.348 |
| Is there a place you go when you need medical care? | 202/245 (82.4%) | 152/177 (85.9%) | 30/40 (75.0%) | 20/28 (71.4%) | 0.070 |
| What type of area is your health care provider?  Urban  Suburban  Rural | Total: 244      115 (47.1%)  103 (42.2%)  26 (10.7%) | Total: 178      80 (44.9%)  75 (42.1%)  23 (12.9%) | Total: 39      17 (43.6%)  21 (53.8%)  1 (2.3%) | Total: 27      18 (66.7%)  7 (25.9%)  2 (7.4%) | 0.063 |
| How long does it take for you to get to your healthcare provider?  0-10 mins  10-30 mins  30 – 1 hour  Over 1 hour | Total: 246      54 (22.0%)  131 (53.3%)  37 (15.0%)  24 (9.8%) | Total: 179      44 (24.6%)  92 (51.4%)  24 (13.4%)  19 (10.6%) | Total: 39      6 (15.4%)  28 (71.8%)  4 (10.3%)  1 (2.6%) | Total: 28      4 (14.3%)  11 (39.3.%)  9 (32.1%)  4 (14.2%) | 0.021 |
| Which professionals have you talked to in the last year?  Hematologist  General care doctor  NP,PA, midwife  ObGYN  Mental Health Prof  Eye doctor  Podiatrist  Chiropractor  PT, ST, OT  Dentist  Healer | Total: 247      180 (72.9%)  199 (80.6%)  109 (44.1%)  62 (25.1%)  66 (26.7%)  114 (46.2%)  17 (6.9%)  29 (11.7%)  32 (13.0%)  115 (46.6%)  13 (5.3%) | Total: 179      128 (71.5%)  145 (81.0%)  80 (44.7%)  46 (25.7%)  56 (31.3%)  84 (46.9%)  12 (6.7%)  25 (14.0%)  26 (14.5%)  88 (49.2%)  11 (6.1%) | Total: 40      32 (80.0%)  29 (72.5%)  15 (37.5%)  10 (25.5%)  7 (17.5%)  17 (42.5%)  2 (5.0%)  1 (2.5%)  3 (7.5%)  13 (32.5%)  2 (5.0%) | Total: 28      20 (71.4%)  25 (89.3%)  14 (50.0%)  6 (21.4%)  3 (10.7%)  13 (46.4%)  3 (10.7%)  3 (10.7%)  3 (10.7%)  14 (50.0%)  0 (0.0%) | 0.306  0.582  0.738  0.883  0.042  0.989  0.674  0.155  0.530  0.299  0.409 |
| How often were you treated with respect by your provider?  All of the time  Most of the time  Some of the time  Never | Total: 247      152 (61.5%)  74 (30.0%)  19 (7.7%)  2 (0.8%) | Total: 179      108 (60.3%)  54 (30.2%)  16 (8.9%)  1 (0.6%) | Total: 40      27 (67.5%)  9 (22.5%)  3 (7.5%)  1 (2.5%) | Total: 28      17 (60.7%)  9 (32.1%)  3 (10.7%)  1 (3.6%) | 0.409 |
| How often were you asked about your beliefs on healthcare?  All of the time  Most of the time  Some of the time  Never | Total: 243      52 (21.4%)  71 (29.2%)  72 (29.6%)  48 (19.8%) | Total: 177      36 (20.3%)  51 (28.8%)  55 (31.1%)  35 (19.8%) | Total: 38      10 (26.3%)  12 (31.6%)  7 (18.4%)  9 (23.7%) | Total: 28      6 (21.4%)  8 (28.6%)  10 (35.7%)  4 (14.3%) | 0.755 |
| How often was information from your doctor easy to understand?  All of the time  Most of the time  Some of the time  Never | Total: 247      90 (36.4%)  112 (45.3%)  41 (16.6%)  4 (1.6%) | Total: 179      63 (35.2%)  84 (46.9.%)  29 (16.2%)  3 (1.7%) | Total: 40      18 (45.0%)  14 (35.0%)  7 (17.5%)  1 (2.5%) | Total: 28      9 (32.1%)  14 (50.0%)  5 (17.9%)  0 (0.0%) | 0.820 |
| Have you delayed medical care for any of the following reasons?  No transportation  Rural  Nervous  No time off work  No childcare  No adult care  Couldn’t afford copay  Deductible too high  Pay out of pocket  Other | Total: 247        16 (6.5%)  10 (4.0%)  54 (21.9%)  29 (11.7%)  3 (1.2%)  1 (0.4%)  25 (10.1%)  20 (8.1%)  42 (17.0%)  62 (25.1%) | Total: 179        10 (5.6%)  4 (2.2%)  42 (23.5%)  24 (13.4%)  1 (0.6%)  0  17 (9.5%)  13 (7.3%)  23 (12.8%)  44 (24.6%) | Total: 40        4 (10.0%)  3 (7.5%)  7 (17.5%)  4 (10.0%)  1 (2.5%)  0  5 (12.5%)  5 (12.5%)  10 (25.0%)  10 (25.0%) | Total: 28        2 (7.1%)  3 (10.7%)  5 (17.9%)  1 (3.6%)  1 (3.6%)  1 (3.6%)  3 (10.7%)  2 (7.1%)  9 (32.1%)  8 (28.6%) | 0.514  0.045  0.709  0.328  0.274  0.020  0.775  0.458  0.011  0.880 |
| What is your employment status?  Working full time  Working part time  Student  Homemaker  Unemployed  Unemployed bc of TTP | Total: 240    133 (55.4%)  32 (13.3%)  4 (1.7%)  16 (6.7%)  17 (7.1%)  38 (15.8%) | Total: 172    94 (54.6%)  27 (15.7%)  3 (1.7%)  11 (6.4%)  13 (7.6%)  24 (14.0%) | Total: 40    22 (55.0%)  4 (10.0%)  1 (2.5%)  3 (7.5%)  3 (7.5%)  7 (17.5%) | Total: 28    17 (60.7%)  1 (3.6%)  0 (0.0%)  2 (7.1%)  1 (3.6%)  7 (25.0%) | 0.779 |
| What is your current income?  0-50k  50-100k  100-150k  More than 150k  Prefer not to answer | Total: 242    108 (44.6%)  56 (23.1%)  19 (7.9%)  11 (4.5%)  48 (19.8%) | Total: 174    72 (41.4%)  42 (24.1%)  14 (8.0%)  9 (5.2%)  37 (21.3%) | Total: 40    21 (52.5%)  6 (15.0%)  4 (10.0%)  2 (5.0%)  7 (17.5%) | Total: 28    15 (53.6%)  8 (28.6%)  1 (3.6%)  0 (0.0%)  4 (14.3%) | 0.635 |
| Has there been a time when you needed something and could not get it?  Prescription medicine  Mental Health care  ER care  Dental care  Eyeglasses  To see a reg doctor  To see specialist  Follow up | Total: 242        41 (16.9%)  37 (15.3%)  15 (6.2%)  49 (20.2%)  39 (16.1%)  19 (7.9%)  32 (13.2%)  24 (9.9%) | Total: 174        25 (14.4%)  20 (11.5%)  8 (4.6%)  29 (16.7%)  24 (13.8%)  10 (5.7%)  15 (8.6%)  12 (6.9%) | Total: 40        11 (27.5%)  5 (12.5%)  4 (10.0%)  12 (30.0%)  6 (15.0%)  5 (12.5%)  8 (20.0%)  6 (15.0%) | Total: 28        5 (17.9%)  12 (42.9%)  3 (10.7%)  8 (28.6%)  9 (32.1%)  4 (14.3%)  9 (32.1%)  6 (21.4%) | 0.075  0.000  0.199  0.046  0.043  0.107  0.001  0.020 |
| How worried about you about paying your medical bills?  Very worried  Moderately worried  Slightly worried  Not worried at all | Total: 244      63 (25.8%)  56 (23.0%)  54 (22.1%)  71 (29.1%) | Total: 177      41 (23.2%)  43 (24.3%)  37 (20.9%)  56 (31.6%) | Total: 39      9 (34.2%)  5 (12.8%)  13 (33.3%)  12 (30.8%) | Total: 28      13 (46.4%)  8 (28.6%)  4 (14.3%)  3 (10.7%) | 0.027 |
| Have you skipped medication to save money? | 34/243 (14.0%) | 21/176 (11.9%) | 8/39 (20.5%) | 5/28 (17.9%) | 0.309 |
| Have you delayed a prescription to save money? | 38/243 (15.6%) | 25/175 (14.3%) | 7/40 (17.5%) | 6/28 (21.4%) | 0.589 |
| Have you gotten medicine from another country? | 4/243 (1.6%) | 3/176 (1.7%) | 1/38 (2.6%) | 0/28 (0.0%) | 0.706 |
| Have you used alternative therapies to save money? | 35/242 (14.5%) | 23/174 (13.2%) | 7/40 (17.5%) | 7/28 (25.0%) | 0.164 |
| How important is it that your provider understands you?    Very important  Moderately important  Slightly important  Not at all | Total: 242      53 (21.9%)  44 (18.2%)  51 (21.1%)  94 (38.8%) | Total: 174      31 (17.8%)  27 (15.5%)  39 (22.4%)  77 (44.3%) | Total: 40      15 (37.5%)  11 (27.5%)  9 (22.5%)  5 (12.5%) | Total: 28      7 (25.0%)  6 (21.4%)  3 (10.7%)  12 (42.9%) | 0.004 |
| How often were you able to see providers who were similar to you?  Very often  Sometimes  Not often  Never | Total: 231        76 (32.9%)  85 (36.8%)  43 (18.6%)  27 (11.7%) | Total: 166        57 (34.3%)  66 (39.8%)  29 (17.5%)  14 (8.4%) | Total: 39        12 (30.8%)  11 (28.2%)  9 (23.1%)  7 (17.9%) | Total: 26        7 (26.9%)  8 (30.8%)  5 (19.2%)  6 (23.1%) | 0.230 |
| How often have you delayed care due to differences?  Very often  Sometimes  Not often  Never | Total: 244      4 (1.6%)  22 (9.0%)  37 (15.2%)  181 (74.2%) | Total: 176      4 (2.3%)  15 (8.5%)  20 (11.4%)  137 (82.5%) | Total: 40      0 (0.0%)  4 (10.0%)  13 (32.5%)  23 (57.5%) | Total: 28      0 (0.0%)  3 (10.7%)  4 (14.3%)  21 (75.0%) | 0.040 |

Table 8: Differences in medical comorbidities by race

| Category | Total | White | Black | Other | P-value |
| --- | --- | --- | --- | --- | --- |
| Hypertension  No, never  Yes, before TTP  Yes, after TTP | Total: 235  109 (46.4%)  52 (22.1%)  74 (31.5%) | Total: 169  81 (47.9%)  33 (19.5%)  55 (32.5%) | Total: 38  13 (34.2%)  14 (36.8%)  11 (28.9%) | Total: 28  15 (53.6%)  5 (17.9%)  8 (28.6%) | 0.328  0.036  0.972 |
| Lupus  No, never  Yes, before TTP  Yes, after TTP | Total: 217  196 (90.3%)  11 (5.1%)  10 (4.6%) | Total: 155  144 (92.9%)  7 (4.5%)  4 (2.6%) | Total: 36  30 (83.3%)  3 (8.3%)  3 (8.3%) | Total: 26  22 (84.6%)  1 (3.8%)  3 (11.5%) | 0.991  0.530  0.045 |
| Heart Attack  No, never  Yes, before TTP  Yes, after TTP | Total: 213  199 (93.4%)  5 (2.3%)  9 (4.2%) | Total: 156  146 (93.5%)  2 (1.3%)  5 (3.2%) | Total: 35  29 (82.9%)  2 (5.7%)  4 (11.4%) | Total: 25  24 (96.0%)  1 (4.0%)  0 (0.0%) | 0.776  0.209  0.037 |
| Stroke  No, never  Yes, before TTP  Yes, after TTP | Total: 222  157 (70.7%)  11 (5.0%)  54 (24.3%) | Total: 161  113 (70.2%)  9 (5.6%)  39 (24.2%) | Total: 36  24 (66.7%)  1 (2.8%)  11 (30.6%) | Total: 25  20 (80.0%)  1 (4.0%)  4 (16.0%) | 0.720  0.797  0.357 |
| Headaches  No, never  Yes, before TTP  Yes, after TTP | Total: 236  55 (23.3%)  101 (42.8%)  80 (33.9%) | Total: 168  45 (26.8%)  70 (41.7%)  53 (31.5%) | Total: 41  8 (19.5%)  17 (41.5%)  16 (39.0%) | Total: 27  2 (7.4%)  14 (51.8%)  11 (40.7%) | 0.116  0.489  0.232 |
| Depression  No, never  Yes, before TTP  Yes, after TTP | Total: 237  90 (38.0%)  52 (21.9%)  95 (40.1%) | Total: 168  65 (38.6%)  38 (22.6%)  65 (38.6%) | Total: 41  17 (41.5%)  8 (19.5%)  16 (39.0%) | Total: 28  8 (28.6%)  6 (21.4%)  14 (50.0%) | 0.394  0.999  0.349 |
| Do you believe your life is better?  Much worse  Slightly worse  The same  Slightly better  Much better | Total: 241    104 (43.2%)  93 (38.6%)  32 (13.3%)  10 (4.1%)  2 (0.8%) | Total: 173    70 (40.5%)  67 (38.7%)  26 (15.0%)  8 (4.6%)  2 (1.2%) | Total: 40    17 (42.5%)  17 (42.5%)  5 (12.5%)  1 (2.5%)  0 (0.0%) | Total: 28    17 (60.7%)  9 (32.1%)  1 (3.6%)  1 (3.6%)  0 (0.0%) | 0.611 |

**Analyses for differences by geographic area**

Table 9: Differences in sociodemographic information by geographic information

| Category | Total Cohort | Rural | Suburban | Urban | P value |
| --- | --- | --- | --- | --- | --- |
| Age Median (IQR) | 47 (28.5- 65.5)  Total: 276 | 47(26-68)  Total: 66 | 48 (28.5-67.5)  Total: 132 | 46.5 (30.25-62.75)  Total: 78 | R: 0.000  S: 0.000  U: 0.000 |
| Sex (female, %) | 253/276 (91.7%) | 60/66 (90.0%) | 125/132 (94.7%) | 68/78 (87.2%) | 0.158 |
| Race (%)  White  Black  Other | Total: 279  205 (73.5%)  43 (15.4%)  31 (11.1%) | Total: 66  58 (87.9%)  3 (4.5%)  5 (7.6%) | Total: 143  93 (69.4%)  31 (23.1%)  10 (7.5%) | Total: 79  54 (68.4%)  9 (11.4%)  16 (20.3%) | 0.000 |
| Highest degree of school  Never attended- 12th  HS Graduate  GED or Equiv  Some college  Associate/ Bachelors  Masters and higher  Refused  Don’t know | Total: 261    5 (1.9%)  34 (13.0%)  6 (2.3%)  35 (13.4%)  104 (39.8%)  75 (28.7%)  0  2 (0.8%) | Total: 61    1 (1.6%)  15 (24.6%)  2 (3.3%)  6 (9.8%)  25 (41.0%)  12 (19.7%)  0  0 | Total: 127    3 (2.4%)  12 (9.4%)  4 (3.1%)  21 (16.5%)  47 (37.0%)  40 (31.5%)  0  0 | Total: 73    1 (1.4%)  7 (9.6%)  0  8 (11.0%)  29 (39.7%)  23 (31.5%)  0  2 (2.7%) | 0.036 |
| Marital Status (%)  Married/ couple  Divorced/Sep/Widow  Never married  Rather not say | Total: 276  182 (65.9%)  34 (12.3%)  55 (19.9%)  5 (1.8%) | Total: 66  46 (69.7%)  9 (13.6%)  9 (13.6%)  2 (3.0%) | Total: 131  91 (69.5%)  14 (10.7%)  25 (19.1%)  1 (0.8%) | Total: 79  45 (57.0%)  11 (13.9%)  21 (26.6%)  2 (2.5%) | 0.403 |

Table 10: Differences in TTP diagnosis, treatment by geographic information

| Category | Total | Rural | Suburban | Urban | P value |
| --- | --- | --- | --- | --- | --- |
| Are you worried about relapse? (%) | 258/279 (92.5%) | 60/66 (90.9%) | 123/134 (91.8%) | 75/79 (94.9%) | 0.603 |
| Has your doctor discussed symptoms of TTP relapse? (%) | 242/279 (86.7%) | 55/66 (83.3%) | 120/134 (89.6%) | 67/79 (84.9%) | 0.398 |
| Has your doctor discussed what to do in a relapse? (%) | 229/278 (82.4%) | 54/65 (83.1%) | 115/134 (85.8%) | 60/79 (75.9%) | 0.186 |
| Treatments  Steroids  Prednisone  Plasma Exchange  Rituximab  Caplacizumab  Other  I don’t know | Total: 278  178 (64.0%)  214 (77.0%)  273 (97.8%)  207 (74.4%)  48 (17.3%)  61 (21.9%)  2 (0.7%) | Total: 65  44 (67.7%)  50 (76.9%)  63 (96.9%)  44 (67.7%)  6 (9.2%)  17 (26.2%)  0 | Total: 134  89 (66.4%)  106(79.1%)  132(98.5%)  103(76.9%)  19 (14.2%)  33 (24.6%)  0 | Total: 79  45 (57.0%)  58 (73.4%)  78 (98.7%)  60 (75.9%)  23 (29.1%)  11 (13.9%)  2 (2.5%) | 0.328  0.624  0.306  0.275  0.003  0.129  0.078 |
| Has your doctor discussed the risk of TTP relapse? (%) | 221/277 (79.8%) | 50/65 (76.9%) | 113/133 (85.0%) | 58/79 (73.4%) | 0.104 |
| Has your doctor discussed tx for relapse prevention? (%) | 125/277 (45.1%) | 27/65 (41.5%) | 68/133 (51.1%) | 30/79 (38.0%) | 0.142 |
| Do you have a TTP doctor? (%) | 232/277 (83.8%) | 55/65 (84.6%) | 113/132 (85.6%) | 64/79 (81.0%) | 0.678 |
| Do you feel your doctor is knowledgeable about TTP? (%) | 227/275 (82.5%) | 48/66 (72.7%) | 116/131 (88.5%) | 63/78 (80.8%) | 0.020 |
| How long does it take to get to your doctor? (%)  0-10 minutes  10-30 minutes  30 minutes- hour  Over 1 hour | Total: 273      36 (13.2%)  97 (35.5%)  64 (23.4%)  76 (27.8%) | Total: 64      3 (4.7%)  9 (14.1%)  18 (28.1%)  34 (53.1%) | Total: 131      18 (13.7%)  67 (51.1%)  28 (21.4%)  18 (13.7%) | Total: 78      15 (19.2%)  21 (26.9%)  18 (23.1%)  24 (30.8%) | 0.000 |
| Type of TTP DR (%)  Hematologist  Heme/Onc  Other/Don’t know | Total: 274  89 (32.5%)  165 (60.2%)  20 (7.3%) | Total: 66  18 (27.3%)  42 (63.6%)  6 (9.1%) | Total: 131  41 (31.3%)  85 (64.9%)  5 (3.8%) | Total: 77  30 (39.0%)  38 (49.4%)  9 (11.7%) | 0.084 |
| Blood Work (%) | 247/276 (89.5%) | 57/65 (87.7%) | 121/133 (91.0%) | 69/78 (88.5%) | 0.732 |
| Freq of labs (%)  More than 3 mo  3 mo  6 mo  1 yr  Less than 1 year | Total: 274  86 (31.4%)  84 (30.7%)  48 (17.5%)  32 (11.7%)  24 (8.8%) | Total: 65  26 (40.0%)  13 (20.0%)  9 (13.8%)  8 (12.3%)  9 (13.8%) | Total: 131  39 (29.8%)  47 (35.9%)  21 (16.0%)  13 (9.9%)  11 (8.4%) | Total: 78  21 (26.9%)  24 (30.8%)  18 (23.1%)  11 (14.1%)  4 (5.1%) | 0.169 |
| Type of Labs (%)  CBC/Platelets  LDH  ADAMTS13  IDK | Total: 274  262 (95.6%)  192 (70.1%)  179 (65.3%)  17 (6.2%) | Total: 65  63 (96.9%)  44 (67.7%)  36 (55.4%)  4 (6.2%) | Total: 131  126(96.2%)  94 (71.8%)  95 (72.5%)  6 (4.6%) | Total: 78  73 (93.6%)  54 (69.2%)  48 (61.5%)  7 (9.0%) | 0.744  0.878  0.058  0.434 |
| Do you feel your doctor listens to your concerns (%)  Always  Sometimes  Often  Never | Total: 274      165 (60.2%)  55 (20.1%)  46 (16.8%)  8 (2.9%) | Total: 66      37 (56.1%)  12 (18.2%)  14 (21.2%)  3 (4.5%) | Total: 130      84 (64.6%)  29 (22.3%)  14 (10.8%)  3 (2.3%) | Total: 78      44 (56.4%)  14 (17.9%)  18 (23.1%)  2 (2.6%) | 0.267 |
| Types of support (%) | 89/276 (32.2%) | 20/65 (30.8%) | 42/133 (31.6%) | 27/78 (34.6%) | 0.864 |
| Depression or Anxiety (%) | 228/276 (82.6%) | 56/66 (84.8%) | 105/132 (79.5%) | 67/78 (85.9%) | 0.432 |
| Referred to Mental health professional (%) | 82/274 (29.9%) | 19/65 (29.2%) | 39/132 (29.5%) | 24/77 (31.2%) | 0.960 |

Table 11: Differences in Healthcare access and Utilization Survey by geographic information

| Category | Total | Rural | Suburban | Urban | P value |
| --- | --- | --- | --- | --- | --- |
| Insurance (%)  None  Private insurance  Medicare  Medicaid  Other  Prefer not to say | Total: 243  17 (7.0%)  133 (54.7%)  33 (13.6%)  16 (6.6%)  39 (16.0%)  5 (2.1%) | Total: 58  6 (10.3%)  30 (51.7%)  7 (12.1%)  7 (12.1%)  6 (10.3%)  2 (3.4%) | Total: 122  6 (3.5%)  76 (62.3%)  17 (13.9%)  5 (4.1%)  17 (13.9%)  1 (0.8%) | Total: 63  5 (7.9%)  27 (42.9%)  9 (14.3%)  4 (6.3%)  16 (25.4%)  2 (3.2%) | 0.116 |
| In the past year, were you told your insurance was not covered? | 32/240 (13.3%) | 9/59 (15.3%) | 16/121 (13.2%) | 7/60 (11.7%) | 0.846 |
| How does coverage compare to a year ago?  Better  Worse  About the same | Total: 237      19 (8.0%)  35 (14.8%)  183 (77.2%) | Total: 57      4 (7.0%)  10 (17.5%)  43 (75.4%) | Total: 121      10 (8.2%)  15 (12.4%)  96 (79.3%) | Total: 59      5 (8.5%)  10 (16.9%)  44 (74.6%) | 0.880 |
| Is there a place you go when you need medical care? | 201/243 (82.7%) | 44/57 (77.2%) | 108/124 (87.1%) | 49/62 (79.0%) | 0.176 |
| What type of area is your health care provider?  Urban  Suburban  Rural | Total: 243      115 (47.3%)  102 (42.0%)  26 (10.7%) | Total: 59      23 (39.0%)  13 (22.0%)  23 (39.0%) | Total: 123      38 (30.9%)  85 (69.1%)  0 | Total: 61      54 (88.5%)  4 (6.6%)  3 (4.9%) | 0.000 |
| How long does it take for you to get to your healthcare provider?  0-10 mins  10-30 mins  30 – 1 hour  Over 1 hour | Total: 244      54 (22.1%)  130 (53.3%)  36 (14.8%)  24 (9.8%) | Total: 59      14 (23.7%)  21 (35.6%)  12 (20.3%)  12 (20.3%) | Total: 123      24 (19.5%)  85 (69.1%)  9 (7.3%)  5 (4.1%) | Total: 62      16 (25.8%)  24 (38.7%)  15 (24.2%)  7 (11.3%) | 0.000 |
| Which professionals have you talked to in the last year?  Hematologist  General care doctor  NP,PA, midwife  ObGYN  Mental Health Prof  Eye doctor  Podiatrist  Chiropractor  PT, ST, OT  Dentist  Healer | Total: 244      179 (73.4%)  198 (81.1%)  108 (44.3%)  61 (25.0%)  65 (26.6%)  114 (46.7%)  16 (6.6%)  29 (11.9%)  32 (13.1%)  114 (46.7%)  12 (4.9%) | Total: 59      42 (71.2%)  44 (74.6%)  33 (55.9%)  13 (22.0%)  18 (30.5%)  27 (45.8%)  5 (8.5%)  13 (22.0%)  8 (13.6%)  31 (52.5%)  6 (10.2%) | Total: 123      97 (78.9%)  100 (81.3%)  53 (43.1%)  33 (26.8%)  35 (28.5%)  61 (49.6%)  7 (5.7%)  11 (8.9%)  18 (14.6%)  55 (44.7%)  3 (2.4%) | Total: 62      40 (64.5%)  54 (87.1%)  22 (35.5%)  15 (24.2%)  12 (19.4%)  26 (41.9%)  4 (6.5%)  5 (8.1%)  6 (9.7%)  28 (45.2%)  3 (4.8%) | 0.006  0.422  0.023  0.559  0.130  0.195  0.762  0.016  0.427  0.371  0.078 |
| How often were you treated with respect by your provider?  All of the time  Most of the time  Some of the time  Never | Total: 245      151 (61.6%)  73 (29.8%)  19 (7.8%)  2 (0.8%) | Total: 59      29 (49.2%)  22 (37.3%)  8 (13.6%)  0 | Total: 124      85 (68.5%)  31 (25.0%)  7 (5.6%)  1 (0.8%) | Total: 62      37 (59.7%)  20 (32.3%)  4 (6.5%)  1 (1.6%) | 0.170 |
| How often were you asked about your beliefs on healthcare?  All of the time  Most of the time  Some of the time  Never | Total: 241      51 (21.1%)  71 (29.5%)  71 (29.5%)  48 (19.9%) | Total: 58      10 (17.2%)  15 (25.9%)  17 (29.3%)  16 (27.6%) | Total: 122      29 (23.8%)  37 (30.3%)  37 (30.3%)  19 (15.6%) | Total: 61      12 (19.7%)  19 (31.1%)  17 (27.9%)  13 (21.3%) | 0.645 |
| How often was information from your doctor easy to understand?  All of the time  Most of the time  Some of the time  Never | Total: 245        89 (36.3%)  111 (45.3%)  41 (16.7%)  4 (1.6%) | Total: 59        22 (37.3%)  25 (42.4%)  10 (16.9%)  2 (3.4 %) | Total: 124        48 (38.7%)  55 (44.4%)  19 (15.3%)  2 (1.6%) | Total: 62        19 (30.6%)  31 (50.0%)  12 (19.4%)  0 | 0. 713 |
| Have you delayed medical care for any of the following reasons?  No transportation  Rural  Nervous  No time off work  No childcare  No adult care  Couldn’t afford copay  Deductible too high  Pay out of pocket  Other | Total: 245        16 (6.5%)  10 (4.1%)  54 (22.0%)  29 (11.8%)  3 (1.2%)  1 (0.4%)  25 (10.2%)  20 (8.2%)  42 (17.1%)  61 (24.9%) | Total: 59        5 (8.5%)  7 (11.9%)  15 (25.4%)  7 (11.9%)  1 (1.7%)  1 (1.7%)  8 (13.6%)  6 (10.2%)  11 (18.6%)  12 (20.3%) | Total: 124        5 (4.0%)  2 (1.6%)  25 (20.2%)  15 (12.1%)  1 (0.8%)  0  13 (10.5%)  10 (8.1%)  21 (16.9%)  31 (25.0%) | Total: 62        6 (9.7%)  1 (1.6%)  14 (22.6%)  7 (11.3%)  1 (1.6%)  0  4 (6.5%)  4 (6.5%)  10 (16.1%)  18 (29.0%) | 0.384  0.002  0.720  0.863  0.868  0.198  0.306  0.634  0.798  0.708 |
| What is your employment status?  Working full time  Working part time  Student  Homemaker  Unemployed  Unemployed bc of TTP | Total: 239    133 (55.6%)  32 (13.4%)  4 (1.7%)  16 (6.7%)  16 (6.7%)  38 (15.9%) | Total: 55    24 (43.6%)  8 (14.5%)  1 (1.8%)  5 (9.1%)  5 (9.1%)  12 (21.8%) | Total: 122    77 (63.1%)  13 (10.7%)  3 (2.5%)  9 (3.5%)  6 (4.9%)  14 (11.5%) | Total: 62    32 (51.6%)  11 (17.7%)  0  2 (3.2%)  5 (8.1%)  12 (19.35%) | 0.258 |
| What is your current income?  0-50k  50-100k  100-150k  More than 150k  Prefer not to answer | Total: 240    108 (45.0%)  56 (23.3%)  18 (7.5%)  11 (4.6%)  47 (19.6%) | Total: 58    36 (62.0%)  11 (19.0%)  0  2 (3.4%)  9 (15.5%) | Total: 121    46 (38.0%)  26 (21.5%)  13 (10.7%)  8 (6.6%)  28 (23.1%) | Total: 61    26 (42.6%)  19 (31.3%)  5 (8.2%)  1 (1.6%)  10 (16.4%) | 0.026 |
| Has there been a time when you needed something and could not get it?  Prescription medicine  Mental Health care  ER care  Dental care  Eyeglasses  To see a reg doctor  To see specialist  Follow up | Total: 240        41 (17.1%)  37 (15.4%)  15 (6.3%)  49 (20.4%)  39 (16.3%)  19 (7.9%)  32 (13.3%)  24 (10.0%) | Total: 58        13 (22.4%)  11 (19.0%)  4 (6.9%)  12 (20.7%)  13 (22.4%)  6 (10.3%)  11 (19.0%)  6 (10.3%) | Total: 121        22 (18.2%)  17 (14.0%)  8 (6.6%)  28 (23.4%)  13 (22.4%)  9 (7.4%)  16 (13.2%)  14 (11.6%) | Total: 61        6 (9.8%)  9 (14.8%)  3 (4.9%)  9 (14.8%)  13 (21.3%)  4 (6.6%)  5 (8.2%)  4 (6.6%) | 0.090  0.624  0.763  0.210  0.120  0.630  0.147  0.395 |
| How worried about you about paying your medical bills?  Very worried  Moderately worried  Slightly worried  Not worried at all | Total: 242      62 (25.6%)  56 (23.1%)  54 (22.3%)  70 (28.9%) | Total: 58      18 (31.0%)  13 (22.4%)  12 (20.7%)  15 (25.9%) | Total: 122      29 (23.8%)  28 (23.0%)  28 (23.0%)  37 (30.3%) | Total: 62      15 (24.2%)  15 (24.2%)  14 (22.6%)  18 (29.0%) | 0.972 |
| Have you skipped medication to save money? | 34/241 (14.1%) | 9/58 (15.5%) | 14/123 (11.4%) | 11/60 (18.3%) | 0.420 |
| Have you delayed a prescription to save money? | 38/241 (15.8%) | 12/57 (21.1%) | 17/123 (13.8%) | 9/61 (14.8%) | 0.450 |
| Have you gotten medicine from another country? | 4/240 (1.7%) | 1/58 (1.7%) | 1/121 (0.8%) | 2/61 (3.3%) | 0.475 |
| Have you used alternative therapies to save money? | 35/240 (14.6%) | 11/57 (19.3%) | 13/123 (10.6%) | 11/60 (18.3%) | 0.193 |
| How important is it that your provider understands you?  Very important  Moderately important  Slightly important  Not at all | Total: 240      52 (21.7%)  44 (18.3%)  51 (21.3%)  93 (38.8%) | Total: 56      14 (25.0%)  5 (8.9%)  13 (23.2%)  24 (42.9%) | Total: 123      24 (19.5%)  27 (22.0%)  23 (18.7%)  49 (39.8%) | Total: 61      14 (23.0%)  12 (19.7%)  15 (24.6%)  20 (32.8%) | 0.437 |
| How often were you able to see providers who were similar to you?  Very often  Sometimes  Not often  Never | Total: 230        75 (32.6%)  85 (37.0%)  43 (18.7%)  27 (11.7%) | Total: 56        17 (30.4%)  22 (39.3%)  13 (23.2%)  4 (7.1%) | Total: 116        40 (34.5%)  43 (37.1%)  19 (16.4%)  14 (12.1%) | Total: 58        18 (31.0%)  20 (34.5%)  11 (19.0%)  9 (15.5%) | 0.796 |
| How often have you delayed care due to differences?  Very often  Sometimes  Not often  Never | Total: 242    4 (1.7%)  22 (9.1%)  37 (15.3%)  179 (74.0%) | Total: 58    1 (1.7%)  4 (6.9%)  14 (24.1%)  39 (67.2%) | Total: 123    2 (1.6%)  9 (7.3%)  16 (13.0%)  96 (78.0%) | Total: 61    1 (1.6%)  9 (14.8%)  7 (11.5%)  44 (72.1%) | 0.276 |

Table 12: Differences healthcare comorbidities by geographic information

| Category | Total | Rural | Suburban | Urban | P-value |
| --- | --- | --- | --- | --- | --- |
| Hypertension  No, never  Yes, before TTP  Yes, after TTP | Total: 233  108 (46.4%)  51 (21.9%)  74 (31.8%) | Total: 57  27 (47.4%)  12 (21.1%)  18 (31.6%) | Total: 117  49 (41.9%)  30 (25.6%)  38 (23.4%) | Total: 59  32 (54.2%)  9 (15.3%)  18 (30.5%) | 0.778  0.134  0.665 |
| Lupus  No, never  Yes, before TTP  Yes, after TTP | Total: 216  195 (90.3%)  11 (5.1%)  10 (4.6%) | Total: 55   50 (90.9%)  1 (1.8%)  4 (7.3%) | Total:  106  95 (89.6%)  6 (5.7%)  5 (4.7%) | Total: 55  50 (90.9%)  4 (7.3%)  1 (1.8%) | 0.249  0.499  0.300 |
| Heart Attack  No, never  Yes, before TTP  Yes, after TTP | Total: 212  198 (93.4%)  5 (2.4%)  9 (4.2%) | Total: 55  48 (87.3%)  2 (3.6%)  5 (9.1%) | Total: 105  100 (95.2%)  2 (1.9%)  3 (2.9%) | Total: 52  50 (96.2%)  1 (1.9%)  1 (1.9%) | 0.199  0.681  0.068 |
| Stroke  No, never  Yes, before TTP  Yes, after TTP | Total: 221  156 (70.6%)  11 (5.0%)  54 (24.4%) | Total: 57  36 (63.2%)  2 (3.5%)  19 (33.3%) | Total: 109  77 (70.6%)  8 (7.3%)  24 (22.0%) | Total: 55  43 (78.2%)  1 (1.8%)  11 (20.0%) | 0.882  0.213  0.066 |
| Headaches  No, never  Yes, before TTP  Yes, after TTP | Total: 235  55 (23.4%)  100 (42.6%)  80 (34.0%) | Total: 59  12 (20.3%)  24 (40.7%)  23 (39.0%) | Total: 119  26 (21.8%)  56 (47.1%)  37 (31.1%) | Total: 57  17 (29.8%)  20 (35.1%)  20 (35.1%) | 0.874  0.053  0.419 |
| Depression  No, never  Yes, before TTP  Yes, after TTP | Total: 235  90 (38.3%)  52 (22.1%)  93 (39.6%) | Total: 59  19 (32.2%)  16 (27.1%)  24 (40.7%) | Total: 113  46 (40.7%)  25 (22.1%)  42 (37.2%) | Total: 63  25 (39.7%)  11 (17.5%)  27 (42.9%) | 0.726  0.283  0.765 |
| Do you believe your life is better?  Much worse  Slightly worse  The same  Slightly better  Much better | Total: 239    104 (43.5%)  93 (38.9%)  30 (12.6%)  10 (4.2%)  2 (0.8%) | Total: 58    27 (46.6%)  18 (31.0%)  10 (17.2%)  3 (5.2%)  0 | Total: 121    51 (42.1%)  51 (42.1%)  15 (12.4%)  3 (2.5%)  1 (0.8%) | Total: 60    26 (43.3%)  24 (40.0%)  5 (8.3%)  4 (6.7%)  1 (1.7%) | 0.630 |

Analyses for differences by Insurance Status

Table 13: Differences in Sociodemographic information by insurance status

| Category | Total | None | Private | Medicare | Medicaid | Other | Prefer not to say | P value |
| --- | --- | --- | --- | --- | --- | --- | --- | --- |
| Age  Median (IQR) | 47 (28-66)  T: 242 | 41(28-54)  T:18 | 47(30.5-63.5)  T:133 | 66.5(51-82)  T:34 | 37 (17.3-56.8)  T:16 | 43.5 (25-62)  T:39 | 40 (22-58)  T:5 |  |
| Sex (female, %) | 223/242 (92.1%) | 17/18 (94.4%) | 124/132 (82.4%) | 28/34 (82.4%) | 16/16 (100%) | 34/37 (91.9%) | 4/5 (80.0%) | 0.179 |
| Race (%)  White  Black  Other | T: 245  178 (72.7%)  39 (15.9%)  28 (11.4%) | T: 18  9 (50.0%)  5 (2.8%)  4 (2.2%) | T: 133  100 (75.2%)  19 (14.3%)  14 (10.5%) | T: 34  30 (88.2%)  4 (11.8%)  0 | T: 16  10 (62.5%)  3 (18.8%)  3 (18.8%) | T: 39  26 (66.7%)  7 (17.9%)  6 (15.4%) | T:5  3 (60.0%)  1 (20.0%)  1 (20.0%) | 0.230 |
| Highest degree of school  Never attended-12th  HS Graduate  GED or Equiv  Some college  Associate or Bachelor  Masters or higher  Refused  Don’t know | T: 232  4 (1.7%)  33(14.2%)  6 (2.6%)  28 (12.1%)  91 (39.25)  69 (29.7%)  0  1 (0.4%) | T: 17  2 (11.8%)  4 (24.5%)  2 (11.8%)  4 (24.5%)  4 (24.5%)  1 (5.9%)  0  0 | T: 126  2 (1.6%)  13 (10.3%)  3 (2.4%)  15 (11.9%)  55 (43.7%)  38 (30.2%)  0  0 | T: 33  0  6 (18.2%)  1 (3.0%)  3 (9.1%)  10 (30.3%)  13 (39.4%)  0  0 | T: 15  0  3 (20.0%)  0  2 (13.3%)  7 (47.7%)  3 (20.0%)  0  0 | T: 37  0  6 (16.2%)  0  3 (8.1%)  13 (35.1%)  14 (37.8%)  0  1 (2.7%) | T: 4  0  1 (25.0%)  0  1 (25.0%)  2(50.0%)  0  0  0 | 0.162 |
| Marital Status (%)  Married/Couple  Divorce/Sep/Widow  Never married  Rather not say | T: 242  157 (64.9%)  32 (13.2%)  49 (20.2%)  4 (1.7%) | T: 18  10(55.6%  1 (5.6%)  6 (33.3%)  1 (5.6%) | T: 131  91 (69.5%)  17 (13.0%)  22 (16.8%)  1 (0.8%) | T: 34  25 (73.5%)  3 (8.8%)  5 (14.7%)  1 (2.9%) | T: 16  5 (31.3%)  4 (25.0%)  6 (37.5%)  1 (6.3%) | T: 38  23 (60.5%)  6 (15.8%)  1 (2.6%)  0 | T: 5  3 (60.0%)  1 (20.0%)  1 (20.0%)  0 | 0.001 |
| Adults in home Median (IQR) | 1(0)  T: 242 | 2(1)  T:18 | 1(1)  T:133 | 1(1)  T:34 | 1(1)  T:16 | 1(1)  T:39 | 1(1)  T:5 |  |
| Children in home median (IQR) | 1(0)  T: 242 | 1(1) | 0(1) | 0(0) | 0(1) | 0(1) | 0(1) |  |
| Type of living (%)  Rural  Sub-urban  Urban | T: 243  58 (11.5%)  122 (50.2%)  63 (25.9%) | T: 17  6 (35.3%)  6 (35.3%)  5 (14.3%) | T: 133  30 (22.6%)  76 (57.1%)  27 (20.3%) | T: 33  7 (21.2%)  17 (51.5%)  9 (27.3%) | T: 16  7 (43.8%)  5 (31.3%)  4 (25.0%) | T: 39  6 (15.4%)  17 (43.6%)  16 (41.0%) | T: 5  2 (40.0%)  1 (20.0%)  2 (40.0%) | 0.116 |

Table 14: Differences in TTP diagnosis, therapy by insurance status

| Category | Total | None | Private | Medicare | Medicaid | Other | Prefer not to say | P value |
| --- | --- | --- | --- | --- | --- | --- | --- | --- |
| Year of TTP dx Median (IQR) | 2015 (10yrs)  T: 242 | 2012 (11.5yrs)  T: 18 | 2015 (10yrs)  T: 133 | 2013 (8.5yr)  T: 34 | 2015 (8yr)  T: 16 | 2016 (11yr)  T: 39 | 2021 (3yr)  T: 5 |  |
| TTP Episodes Median (IQR) | 1(3)  T: 242 | 1.5 (3.75)  T:18 | 1(2.5)  T:133 | 1(2.75)  T: 34 | 3(3.5)  T: 16 | 1(2.5)  T:39 | 0(4)  T:5 |  |
| Are you worried about relapse? (%) | 226/245  (92.2%) | 17/18  (94.4%) | 123/ 133  (92.5%) | 30/34  (88.2%) | 15/16  (93.8%) | 36/39  (92.3%) | 5/5  (100.0%) | 0.928 |
| Has your doctor discussed symptoms of TTP relapse? (%) | 214/245  (87.3%) | 16/18  (88.9%) | 119/ 133  (89.5%) | 28/34  (82.4%) | 15/16  (93.8%) | 32/39  (82.1%) | 4/5  (80.0%) | 0.673 |
| Has your doctor discussed what to do in a relapse? (%) | 203/244  (83.2%) | 15/18  (83.3%) | 115/ 133  (86.5%) | 28/34  (82.4%) | 10/15  (66.7%) | 31/39  (79.5%) | 4/5  (80.0%) | 0.495 |
| Treatments  Steroids  Prednisone  Plasma Exchange  Rituximab  Caplacizumab  Other  I don’t know | Total: 244  160 (65.6%)  201 (82.4%)  242 (99.2%)  187 (76.6%)  43 (17.6%)  54 (22.1%)  1 (0.4%) | Total: 18  12 (66.7%)  16 (88.9%)  18 (100.0%)  16 (88.9%)  4 (22.2%)  5 (27.8%)  1 (5.6%) | Total: 133  92 (69.2%)  111 (83.5%)  131 (98.5%)  98 (73.7%)  20 (15.0%)  31 (23.3%)  0 | Total: 34  14 (41.2%)  26 (76.5%)  34 (100.0%)  25 (73.5%)  7 (20.6%)  5 (14.7%)  0 | Total: 16  12 (75.0%)  14 (87.5%)  16(100.0%)12 (75.0%)  2 (12.5%)  1 (6.3%)  0 | Total: 39  26 (66.7%)  30 (76.9%)  39 (100.0%)  33 (84.6%)  9 (23.1%)  9 (23.1%)  0 | Total:5  4 (80.0%)  4 (80.0%)  4 (80.0%)  3 (60.0%)  1 (20.0%)  3 (60.0%)  0 | 0.055  0.776  0.007  0.484  0.822  0.152  0.027 |
| Has your doctor discussed the risk of TTP relapse? (%) | 195/244  (79.9%) | 14/18  (77.8%) | 108/ 133  (81.2%) | 22/33  (95.7%) | 15/16  (93.8%) | 32/39  (82.1%) | 4/5  (80.0%) | 0.324 |
| Has your doctor discussed tx for relapse prevention? (%) | 112/244  (45.9%) | 13/18  (72.2%) | 56/ 132  (42.4%) | 15/34  (44.1%) | 11/16  (68.8%) | 17/39  (43.6%) | 0/5 | 0.020 |
| Do you have a TTP doctor? (%) | 205/243  (84.4%) | 17/18  (94.4%) | 112/ 132  (84.8%) | 27/33  (81.8%) | 13/16  (81.3%) | 31/39  (79.5%) | 5/5  (100.0%) | 0.651 |
| Do you feel your doctor is knowledgeable about TTP? (%) | 203/242  (83.9%) | 14/17  (82.4%) | 113/131  (86.3%) | 26/34  (76.5%) | 11/16  (68.8%) | 35/39  (89.7%) | 4/5  (80.0%) | 0.335 |
| How long does it take to get to your doctor? (%)  0-10 minutes  10-30 minutes  30 minutes- hour  Over 1 hour | T: 241  33 (13.7%)  87 (36.1%)  55 (22.8%)  66 (27.4%) | T: 18  0  5 (27.8%)  6 (33.3%)  7 (38.9%) | T:131  21 (16.0%)  50 (38.2%)  29 (22.1%)  31 (23.7%) | T: 32  4 (12.5)  15 (46.9%)  4 (12.5%)  9 (28.1%) | T: 16  1 (6.3%)  4 (25.0%)  2 (12.5%)  9 (56.3%) | T: 39  7 (17.9%)  12 (30.8%)  11 (28.2%)  9 (23.1%) | T: 5  0  1 (20.0%)  3 (60.0%)  1 (20.0%) | 0.135 |
| Type of TTP DR (%)  Hematologist  Heme/Onc  Other/Don’t know | T: 242  77 (31.8%)  150 (61.9%)  15 (6.2%) | T: 18  7 (38.9%)  10 (55.6%)  1 (5.6%) | T:132  40 (30.3%)  83 (62.9%)  9 (6.8%) | T: 35  9 (25.7%)  24 (68.6%)  0 | T:16  6 (37.5%)  8 (66.7%)  2 (12.5%) | T: 38  14 (36.8%)  21 (55.3%)  3 (7.9%) | T: 5  1 (20.0%)  4 (80.0%)  0 | 0.764 |
| Blood Work (%) | 218/244 | 18/18 | 116/ 132 | 30/34 | 13/16 | 36/39 | 5/5 | 0.474 |
| Freq of labs (%)  More than 3 mo  3 mo  6 mo  1 yr  Less than 1 year | T: 243  77 (31.7%)  76 (31.3%)  40 (16.5%)  28 (11.5%)  22 (9.1%) | T:18  4 (22.2%)  6 (33.3%)  4 (22.2%)  3 (16.7%)  1 (5.6%) | T: 132  45 (34.1%)  44 (33.3%)  16 (12.1%)  17 (12.9%)  10 (7.6%) | T:34  11 (32.4%)  5 (14.7%)  8 (23.5%)  4 (11.8%)  6 (17.6%) | T: 15  0  10 (66.7%)  1 (6.7%)  1 (6.7%)  3 (20.0%) | T: 39  16 (41.0%)  9 (23.1%)  9 (23.1%)  3 (7.7%)  2 (5.1%) | T: 5  1 (20.0%)  2 (40.0%)  2 (40.0%)  0  0 | 0.044 |
| Type of Labs (%)  CBC/Platelets  LDH  ADAMTS13  IDK | Total: 243  232 (95.4%)  170 (70.0%)  161 (66.3%)  14 (5.8%) | Total:18  18 (100.0%)  12 (66.7%)  12 (66.7%)  0 | Total: 132  127 (96.2%)  93 (70.5%)  91 (68.9%)  4 (3.0%) | Total: 34  32 (94.1%)  21 (61.8%)  21 (61.8%)  3 (8.8%) | Total: 15  14 (93.3%)  10 (66.7%)  11 (73.3%)  2 (13.3%) | Total: 39  37 (94.9%)  30 (76.9%)  23 (59.0%)  3 (7.7%) | Total: 5  4 (80.0%)  4 (80.0%)  3 (60.0%)  2 (40.0%) | 0.416  0.750  0.901  0.007 |
| Do you feel your doctor listens to your concerns (%)  Always  Sometimes  Often  Never | T: 243  147 (60.4%)  45 (18.5%)  43 (17.7%)  8 (3.3%) | T: 18  10 (55.6%)  4 (22.2%)  4 (22.2%)  0 | T: 131  77 (58.8%)  26 (19.8%)  23 (17.6%)  5 (3.8%) | T: 34  23 (67.6%)  6 (17.6%)  5 (14.7%)  0 | T: 16  9 (56.3%)  3 (18.8%)  3 (18.8%)  1 (6.3%) | T: 39  25 (64.1%)  5 (12.8%)  7 (17.9%)  2 (5.1%) | T: 5  3 (60.0%)  1 (20.0%)  1 (20.0%)  0 | 0.994 |
| Types of support (%) | 82/245  (33.5%) | 8/18  (44.4%) | 45/133  (33.8%) | 10/34  (29.4%) | 5/16  (31.3%) | 13/39  (33.3%) | 1/5  (20.0%) | 0.892 |
| Depression or Anxiety (%) | 204/244  (83.6%) | 16/18  (88.9%) | 115/ 133  (86.5%) | 25/33  (75.8%) | 15/16  (93.8%) | 30/39  (76.9%) | 3/5  (60.0%) | 0.210 |
| Referred to Mental health professional (%) | 77/244  (31.6%) | 6/18  (33.3%) | 41/ 132  (31.1%) | 14/34  (41.2%) | 3/16  (18.8%) | 12/39  (30.8%) | 1/5  (20.0%) | 0.695 |

Table 15: Differences in Healthcare access and utilization survey by insurance status

| Category | Total | None | Private | Medicare | Medicaid | Other | Prefer not to say | P value |
| --- | --- | --- | --- | --- | --- | --- | --- | --- |
| In the past year, were you told your insurance was not covered? | 31/241  (12.9%) | 2/17  (11.8%) | 16/133  (12.0%) | 3/34  (8.8%) | 6/16  (3.8%) | 3/36  (8.3%) | 1/5  (20.0%) | 0.071 |
| How does coverage compare to a year ago?  Better  Worse  Same | T: 238  19 (8.0%)  35 (14.7%)  184 (77.3%) | T: 16  0  7 (43.8%)  9 (56.3%) | T: 132  12 (9.1%)  18 (13.6%)  102 (77.3%) | T: 34  3 (8.8%)  2 (5.9%)  29(85.3%) | T: 16  0  2 (12.5%)  14 (87.5%) | T: 35  2 (5.7%)  5 (14.3%)  28 (80.0%) | T: 5  2 (40.0%)  1 (20.0%)  2 (20.0%) | 0.011 |
| Is there a place you go when you need medical care? | 200/ 243  (82.3%) | 12/18  (66.7%) | 112/ 133  (84.2%) | 30/34  (88.2%) | 12/16  (75.0%) | 30/38  (78.9%) | 4/4  (100.0%) | 0.315 |
| What type of area is your health care provider?  Urban  Suburban  Rural | T: 242  114 (47.1%)  103 (42.6%)  25 (10.3%) | T: 16  11 (68.8%)  2 (12.5%)  3 (18.8%) | T: 133  60 (45.1%)  64 (48.1%)  9 (6.8%) | T: 34  13(38.2.%)16(47.1%)  5(14.7%) | T: 16  8 (50.0%)  5 (31.3%)  3 (18.8%) | T: 38  21 (55.3%)  13 (34.2%)  4 (10.5%) | T: 5  1 (20.0%)  3 (60.0%)  1 (20.0%) | 0.151 |
| How long does it take for you to get to your healthcare provider?  0-10 mins  10-30 mins  30 – 1 hour  Over 1 hour | T:244  54 (22.1%)  131 (53.7%)  35 (14.3%)  24 (9.8%) | T: 18  2 (11.1%)  8 (44.4%)  4 (22.2%)  4 (22.2%) | T: 133  32 (24.1%)  75 (56.4%)  15 (11.3%)  11 (8.3%) | T: 34  6 (17.6%)  20(58.8%)  5 (14.7%)  3 (8.8%) | T: 16  6 (37.5%)  4 (25.0%)  4 (25.0%)  2 (12.5%) | T: 38  7 (18.4%)  21 (55.3%)  6 (15.8%)  4 (10.5%) | T: 5  1 (20.0%)  3 (60.0%)  1 (20.0%)  0 | 0.563 |
| Which professionals have you talked to in the last year?  Hematologist  General care doctor  NP,PA, midwife  ObGYN  Mental Health Prof  Eye doctor  Podiatrist  Chiropractor  PT, ST, OT  Dentist  Healer | Total: 244  178 (73.0%)  196 (80.3%)  107 (43.9%)  62 (25.4%)  65 (26.6%)  114 (46.7%)  16 (6.6%)  28 (11.5%)  32 (13.1%)  115 (47.3%)  11 (4.5%) | Total: 18  11 (61.1%)  11 (61.1%)  4 (22.2%)  2 (11.1%)  3 (16.7%)  5 (27.8%)  0  3 (16.7%)  1 (5.6%)  5 (27.8%)  0 | Total: 133  95 (71.4%)  106 (79.7%)  58 (43.6%)  36 (27.1%)  39 (29.3%)  57 (42.9%)  9 (6.8%)  17 (12.8%)  19 (14.3%)  67 (50.4%)  8 (6.0%) | Total: 34  27(79.4%)  31(91.2%)  19(55.9%)  8 (23.5%)  9 (26.5%)  22(64.7%)  4 (11.8%)  2 (5.9%)  6 (17.6%)  19(55.9%)  1 (2.9%) | Total: 16  12 (75.0%)  10 (62.5%)  10 (62.5%)  4 (25.0%)  4 (25.0%)  6 (37.5%)  1 (6.3%)  2 (12.5%)  2 (12.5%)  7 (43.8%)  1 (6.3%) | Total: 38  29 (76.3%)  33 (86.8%)  13 (34.2%)  10 (26.3%)  9 (23.7%)  22 (57.9%)  2 (5.3%)  4 (10.5%)  4 (10.5%)  13 (34.2%)  1 (2.6%) | Total: 5  4 (80.0%)  5 (100.0%)  3 (60.0%)  2 (40.0%)  1 (20.0%)  2 (40.0%)  0  0  0  4 (80.0%)  0 | 0.802  0.042  0.076  0.736  0.876  0.078  0.658  0.780  0.742  0.090  0.785 |
| How often were you treated with respect by your provider?  All of the time  Most of the time  Some of the time  Never | T: 244  151 (61.9%)  72 (29.5%)  19 (7.8%)  2 (0.8%) | T: 18  9 (50.0%)  6 (33.3%)  3 (16.7%)  0 | T: 133  81 (60.9%)  40 (30.1%)  11 (8.3%)  1 (0.8%) | T: 34  24(70.6%)  9 (26.5%)  1 (2.9%)  0 | T: 16  9 (56.3%)  5 (31.3%)  2 (12.5%)  0 | T: 38  26 (68.4%)  10 (35.7%)  1 (2.6%)  1 (2.6%) | T: 5  2 (40.0%)  2 (40.0%)  1 (20.0%)  0 | 0.829 |
| How often were you asked about your beliefs on healthcare?  All of the time  Most of the time  Some of the time  Never | T: 241  52 (21.6%)  71 (29.5%)  70 (29.0%)  48 (19.9%) | T: 18  6 (33.3%)  5 (27.8%)  4 (22.2%)  3 (16.7%) | T: 132  27 (20.5%)  41 (31.1%)  38 (28.8%)  26 (19.7%) | T: 33  8 (24.2%)  9 (27.3%)  9 (27.3%)  7 (21.1%) | T: 16  5 (31.3%)  5 (31.3%)  4 (25.0%)  2 (12.5%) | T: 37  6 (16.2%)  9 (24.3%)  13 (35.1%)  9 (24.3%) | T: 5  0  2 (40.0%)  2 (40.0%)  1 (20.0%) | 0.973 |
| How often was information from your doctor easy to understand?  All of the time  Most of the time  Some of the time  Never | T: 244  89 (36.5%)  111 (45.5%)  40 (16.4%)  4 (1.6%) | T: 18  7 (38.9%)  5 (27.8%)  4 (22.2%)  2 (11.1%) | T: 133  46 (34.6%)  60 (45.1%)  25 (18.8%)  2 (1.5%) | T: 34  15(44.1%)  15(44.1%)  4 (11.8%)  0 | T: 16  8 (50.0%)  7 (43.8%)  1 (6.3%)  0 | T: 38  12 (31.6%)  22 (57.9%)  4 (10.5%)  0 | T: 5  1 (20.0%)  2 (40.0%)  2 (40.0%)  0 | 0.140 |
| Have you delayed medical care for any of the following reasons?  No transport  Rural  Nervous  No time off  No childcare  No adult care  Couldn’t afford copay  Deductible too high  Pay out of pocket  Other | Total: 244  14 (5.7%)  9 (3.7%)  53 (21.7%)  28 (11.5%)  3 (1.2%)  1 (0.4%)  25 (10.2%)  20 (8.2%)  41 (16.8%)  61 (25.0%) | Total: 18  2 (11.1%)  1 (5.6%)  1 (5.6%)  1 (5.6%)  0  0  5 (27.8%)  4 (22.2%)  8 (44.4%)  3 (16.7%) | Total: 133  4 (3.0%)  3 (2.3%)  30 (22.6%)  18 (13.5%)  2 (1.5%)  0  13 (9.8%)  13 (9.8%)  25 (18.8%)  37 (27.8%) | Total: 34  5 (14.7%)  1 (2.9%)  8 (23.5%)  1 (2.9%)  0  0  2 (5.9%)  1 (2.9%)  0  6 (17.6%) | Total: 16  1 (6.3%)  3 (18.8%)  5 (31.3%)  3 (18.8%)  0  1 (6.3%)  1 (6.3%)  0  2 (12.5%)  4 (25.5%) | Total: 38  1 (2.6%)  1 (2.6%)  7 (18.4%)  4 (10.5%)  1 (2.6%)  0  3 (7.9%)  1 (2.6%)  5 (13.2%)  9 (23.7%) | Total: 5  1 (20.0%)  0  2 (40.0%)  1 (20.0%)  0  0  1 (20.0%)  1 (20.0%)  1 (20.0%)  2 (40.0%) | 0.062  0.041  0.408  0.431  0.905  0.013  0.165  0.064  0.003  0.716 |
| What is your employment status?  Working full time  Working part time  Student  Homemaker  Unemployed  Unemployed bc TTP | T: 238  133 (55.9%)  31 (13.0%)  4 (1.7%)  16 (6.7%)  17 (7.1%)  37 (15.5%) | T: 18  7 (38.8%)  4 (22.2%)  1 (5.6%)  2 (11.1%)  1 (5.6%)  3 (16.7%) | T: 133  94 (70.7%)  12 (9.0%)  2 (1.5%)  9 (6.8%)  4 (3.0%)  12 (9.0%) | T: 30  4 (13.3%)  8 (26.7%)  0  3 (10.0%)  9 (30.0%)  6 (20.0%) | T: 15  4 (26.7%)  4 (26.7%)  0  0  1 (6.7%)  6 (40.0%) | T: 38  23 (60.5%)  2 (5.3%)  1 (2.6%)  2 (5.3%)  1 (2.6%)  9 (23.7%) | T: 4  1 (20.0%)  1 (20.0%)  0  0  1 (20.0%)  1 (20.0%) | 0.000 |
| What is your current income?  0-50k  50-100k  100-150k  > 150k  Prefer not to answer | T: 240  107 (44.6%)  56 (23.3%)  19 (7.9%)  11 (4.6%)  47 (19.6%) | T: 18  13 (72.2%)  2 (11.1%)  0  0  3 (16.7%) | T: 131  44 (33.6%)  39 (29.8%)  15 (11.5%)  9 (6.9%)  24 (18.3%) | T: 34  17(50.0%)  3 (8.8%)  3 (8.8%)  2 (5.9%)  9 (26.5%) | T: 16  13 (81.3%)  0  0  0  3 (18.3%) | T: 36  18 (50.0%)  10 (27.8%)  1 (2.8%)  0  7 (19.4%) | T: 5  2 (40.0%)  2 (40.0%)  0  0  1 (20.0%) | 0.017 |
| Has there been a time when you needed something and could not get it?  Prescription medicine  Mental Health care  ER care  Dental care  Eyeglasses  To see a reg doctor  To see specialist  Follow up | Total: 240  39 (16.3%)  36 (15.0%)  14 (5.8%)  48 (20.0%)  39 (16.3%)  19 (7.9%)  30 (12.5%)  23 (9.6%) | Total: 18  5 (27.8%)  8 (44.4%)  2 (11.1%)  9 (50.0%)  4 (22.2%)  6 (33.3%)  7 (38.9%)  7 (38.9%) | Total: 133  27 (20.3%)  20 (15.0%)  8 (6.0%)  20 (15.0%)  17 (12.8%)  6 (4.5%)  16 (12.3%)  11 (8.3%) | Total: 34  3 (8.8%)  2 (5.9%)  2 (5.9%)  5 (5.9%)  3 (8.8%)  1 (2.9%)  1 (2.9%)  1 (2.9%) | Total: 16  1 (6.3%)  1 (6.3%)  1 (6.3%)  6 (37.5%)  8 (50.0%)  3 (18.8%)  4 (25.0%)  2 (12.5%) | Total: 36  2 (5.6%)  4 (11.1%)  0  7 (19.4%)  5 (13.9%)  2 (5.6%)  2 (5.6%)  1 (2.8%) | Total: 5  1 (20.0%)  1 (20.0%)  1 (20.0%)  1 (20.0%  2 (40.0%)  1 (20.0%)  0  1 (20.0%) | 0.086  0.006  0.385  0.006  0.002  0.000  0.001  0.000 |
| How worried about you about paying your medical bills?  Very worried  Moderately worried  Slightly worried  Not worried at all | T: 243  63 (25.9%)  56 (23.0%)  54 (22.2%)  70 (28.8%) | T: 18  10 (55.6%)  2 (11.1%)  1 (5.6%)  5 (27.8%) | T: 133  37 (27.8%)  36 (27.1%)  32 (24.1%)  28 (21.1%) | T: 34  1 (2.9%)  12(35.3%)  5 (14.7%)  16(47.1%) | T: 16  6 (37.5%)  2 (12.5%)  5 (31.3%)  3 (18.8%) | T: 37  8 (21.6%)  4 (10.8%)  9 (24.3%)  16 (43.2%) | T: 5  1 (20.0%)  0  2 (40.0%)  2 (40.0%) | 0.001 |
| Have you skipped medication to save money? | 34/240  (14.2%) | 3/17  (17.6%) | 20/131  (15.3%) | 1/34  (2.9%) | 2/16  (12.5%) | 4/37  (10.8%) | 2/5  (40.0%) | 0.209 |
| Have you delayed a prescription to save money? | 37/241  (15.4%) | 2/18  (11.1%) | 25/132  (18.9%) | 2/33  (6.1%) | 3/16  (18.8%) | 4/37  (10.8%) | 1/5  (20.0%) | 0.472 |
| Have you gotten medicine from another country? | 4/240  (1.7%) | 0/18 | 4/131  (3.1%) | 0/34 | 0/16 | 0/36 | 0/5 | 0.641 |
| Have you used alternative therapies to save money? | 33/239  (13.8%) | 3/18  (1.7%) | 21/131  (16.0%) | 1/34  (2.9%) | 3/16  (18.8%) | 5/35  (14.3%) | 0/5 | 0.395 |
| How important is it that your provider understands you?  Very important  Moderately important  Slightly important  Not at all | T: 239  53 (22.2%)  41 (17.2%)  51 (21.3%)  94 (39.2%) | T: 18  8 (44.4%)  3 (16.7%)  2 (11.1%)  5 (27.8%) | T: 132  29 (22.0%)  22 (16.7%)  33 (25.0%)  48 (36.4%) | T: 33  7 (21.2%)  5 (15.2%)  7 (21.2%)  14(42.4%) | T: 15  3 (20.0%)  2 (13.3%)  4 (26.7%)  6 (40.0%) | T: 37  5 (13.5%)  9 (24.3%)  5 (13.5%)  18 (48.6%) | T: 4  1 (25.0%)  0  0  3 (75.0%) | 0.504 |
| How often were you able to see providers who were similar to you?  Very often  Sometimes  Not often  Never | T: 228  75 (32.9%)  84 (36.8%)  42 (18.4%)  27 (11.8%) | T: 17  5 (29.4%)  4 (23.5%)  4 (23.5%)  4 (23.5%) | T: 127  42 (33.1%)  51 (40.2%)  21 (16.5%)  13 (10.2%) | T: 29  11(37.9%)  11(37.9%)  7 (17.9%)  0 | T: 15  3 (20.0%)  4 (26.7%)  4 (26.7%)  4 (26.7%) | T: 36  14 (38.9%)  11 (30.6%)  6 (16.7%)  5 (31.3%) | T: 4  0  3 (75.0%)  0  1 (25.0%) | 0.270 |
| How often have you delayed care due to differences?  Very often  Sometimes  Not often  Never | T: 241  4 (1.7%)  21 (8.7%)  35 (14.5%)  181 (75.1%) | T: 18  0  2 (11.1%)  5 (27.8%)  11 (61.1%) | T: 133  3 (2.3%)  12 (9.0%)  22 (16.5%)  96 (72.2%) | T: 33  0  2 (6.1%)  1 (3.0%)  30(90.9%) | T: 16  0  2 (12.5%)  1 (6.3%)  13 (81.3%) | T: 37  1 (2.7%)  2 (5.4%)  6 (16.2%)  28 (75.7%) | T: 4  0  1 (25.0%)  0  3 (75.0%) | 0.617 |

Table 16: Differences in Medical Comorbidities by insurance status

| Category | Total | None | Private | Medicare | Medicaid | Other | Prefer not to say | P-value |
| --- | --- | --- | --- | --- | --- | --- | --- | --- |
| HTN  No, never  Yes, before TTP  Yes, after TTP | T: 233  107 (45.9%)  52 (22.3%)  74 (31.8%) | T:16  10 (6.3%)  3 (18.8%)  3 (18.8%) | T:130  60 (46.2%)  24 (18.5%)  46 (35.4%) | T: 30  9 (30.0%)  13 (43.3%)  8 (26.7%) | T:16  8 (50.0%)  4 (25.0%)  4 (25.0%) | T: 37  18 (48.6%)  8 (21.6%)  11 (29.7%) | T:4  2 (50.0%)  0  2 (50.0%) | 0.345  0.135  0.549 |
| Lupus  No, never  Yes, before TTP  Yes, after TTP | T: 215  194 (90.2%)  11 (5.1%)  10 (4.7%) | T:17  16 (94.1%)  0  1 (5.9%) | T: 122  112(92.8%)  6 (4.9%)  4 (3.3%) | T: 23  21 (91.3%)  1 (4.3%)  1 (4.3%) | T:15  13 (86.7%)  1 (6.7%)  1 (6.7%) | T: 34  29 (85.3%)  3 (8.8%)  2 (5.8%) | T: 4  3 (75.0%)  0  1 (25.0%) | 0.051  0.803  0.529 |
| Heart Attack  No, never  Yes, before TTP  Yes, after TTP | T: 211  197 (93.4%)  5 (2.4%)  9 (4.3%) | T: 17  16 (94.1%)  0  1 (5.9%) | T: 120  111(92.5%)  5 (4.2%)  4 (3.3%) | T:24  22 (91.7%)  0  2 (8.3%) | T: 14  14 (100.0%)  0  0 | T: 33  31 (93.9%)  0  2 (6.1%) | T: 3  3 (100.0%)  0  0 | 0.118  0.507  0.870 |
| Stroke  No, never  Yes, before TTP  Yes, after TTP | T: 220  15 (70.5%)  11 (5.0%)  54 (24.5%) | T:15  13 (86.7%)  0  2 (13.3%) | T: 125  87 (69.6%)  9 (7.2%)  29 (23.2%) | T: 27  16 (59.3%)  1 (3.7%)  10 (37.3%) | T: 15  12 (80.0%)  0  3 (20.0%) | T: 35  24 (68.6%)  1 (2.9%)  10 (28.6%) | T: 3  3 (100.0%)  0  0 | 0.331  0.554  0.530 |
| Headaches  No, never  Yes, before TTP  Yes, after TTP | T: 233  55 (23.6%)  100 (42.9%)  78 (33.5%) | T: 17  5 (29.4%)  7 (41.2%)  5 (29.4%) | T: 130  29 (22.3%)  59 (45.4%)  42 (32.3%) | T: 25  6 (24.0%)  12 (48.0%)  7 (28.0%) | T: 18  1 (5.6%)  7 (38.9%)  10 (55.6%) | T: 38  12 (31.6%)  14 (36.8%)  12 (31.6%) | T: 5  2 (40.0%)  1 (20.0%)  2 (40.0%) | 0.345  0.778  0.100 |
| Depression  No, never  Yes, before TTP  Yes, after TTP | T: 234  90 (38.5%)  49 (20.2%)  95 (39.1%) | T: 18  7 (38.9%)  3 (16.7%)  8 (44.4%) | T: 127  48 (37.8%)  27 (21.3%)  52 (40.0%) | T: 28  12 (42.9%)  7 (25.0%)  9 (32.1%) | T: 17  4 (23.5%)  5 (29.4%)  8 (47.1%) | T: 39  18 (46.2%)  5 (12.8%)  16 (41.0%) | T: 5  1 (20.0%)  2 (40.0%)  2 (40.0%) | 0.680  0.562  0.646 |
| Do you believe your life is better?  Much worse  Slightly worse  The same  Slightly better  Much better | T: 238  104 (43.7%)  90 (37.8%)  32 (13.4%)  10 (4.2%)  2 (0.8%) | T:17  8 (46.1%)  5 (29.4%)  4 (23.5%)  0  0 | T: 131  56 (42.7%)  58 (44.3%)  13 (9.9%)  3 (2.2%)  1 (0.8%) | T: 32  16 (50.0%)  7 (21.9%)  7 (21.9%)  2 (6.3%)  0 | T: 16  8 (50.0%)  2 (12.5%)  3 (18.8%)  3 (18.8%)  0 | T: 38  13 (34.2%)  17 (44.7%)  5 (13.2%)  2 (5.3%)  1 (2.6%) | T: 4  3 (75.0%)  1 (25.0%)  0  0  0 | 0.133 |
